# Supplementary material for: An ancient family of lytic polysaccharide monooxygenases with roles in arthropod development and biomass digestion
Source: Nat Commun. 2018 Feb 22;9:756. doi: 10.1038/s41467-018-03142-x (PMC5823890; doi:10.1038/s41467-018-03142-x)
Supplement: Supplementary file 1 — Supplementary Information [file 41467_2018_3142_MOESM1_ESM.pdf]

Supplementary Information for:

An ancient family of lytic polysaccharide monooxygenases with roles in arthropod development and biomass digestion

Sabbadin *et al.*

Supplementary Information

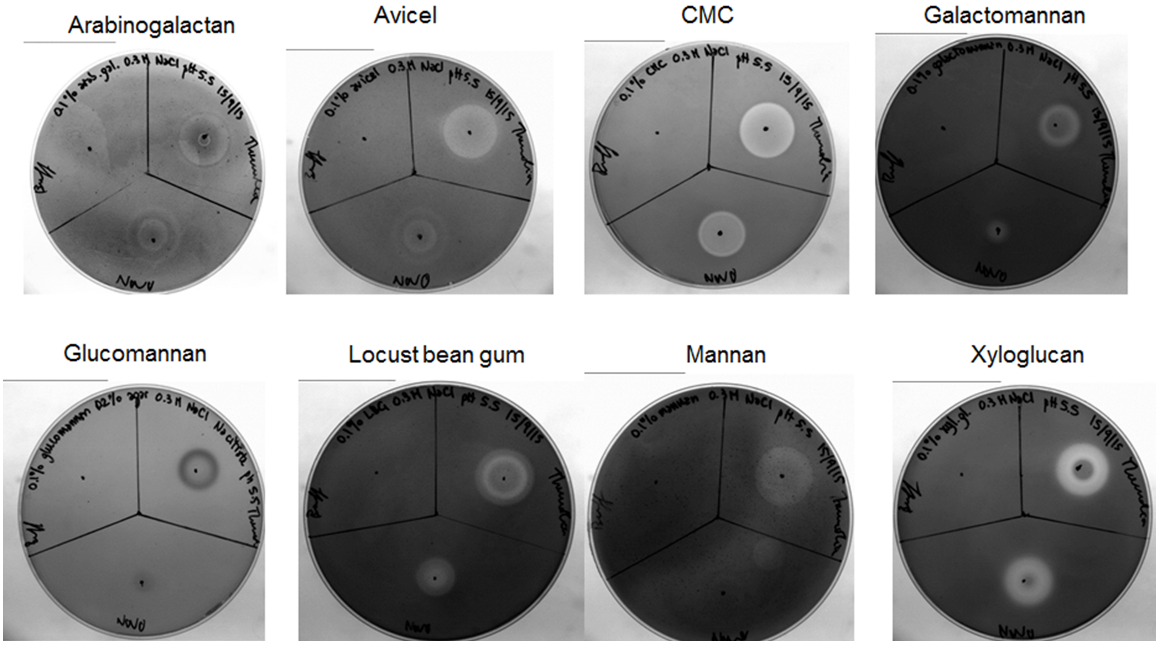

Supplementary Figure 1 | Agar plate assays with soluble protein extract from *T. domestica*`s crop.

Activity assays were carried out on agar plates containing 0.1 % substrate and stained with Congo Red. Each plate was divided in three sectors, spotted either with buffer control (top left), *T. domestica* crop extract (5.6 µg of soluble protein, top right) and positive control (1/100 dilution of Celluclast®, bottom). Clearance zones are visible on all substrates, indicating degradation of the polysaccharides by the protein extract of *Thermobia*.

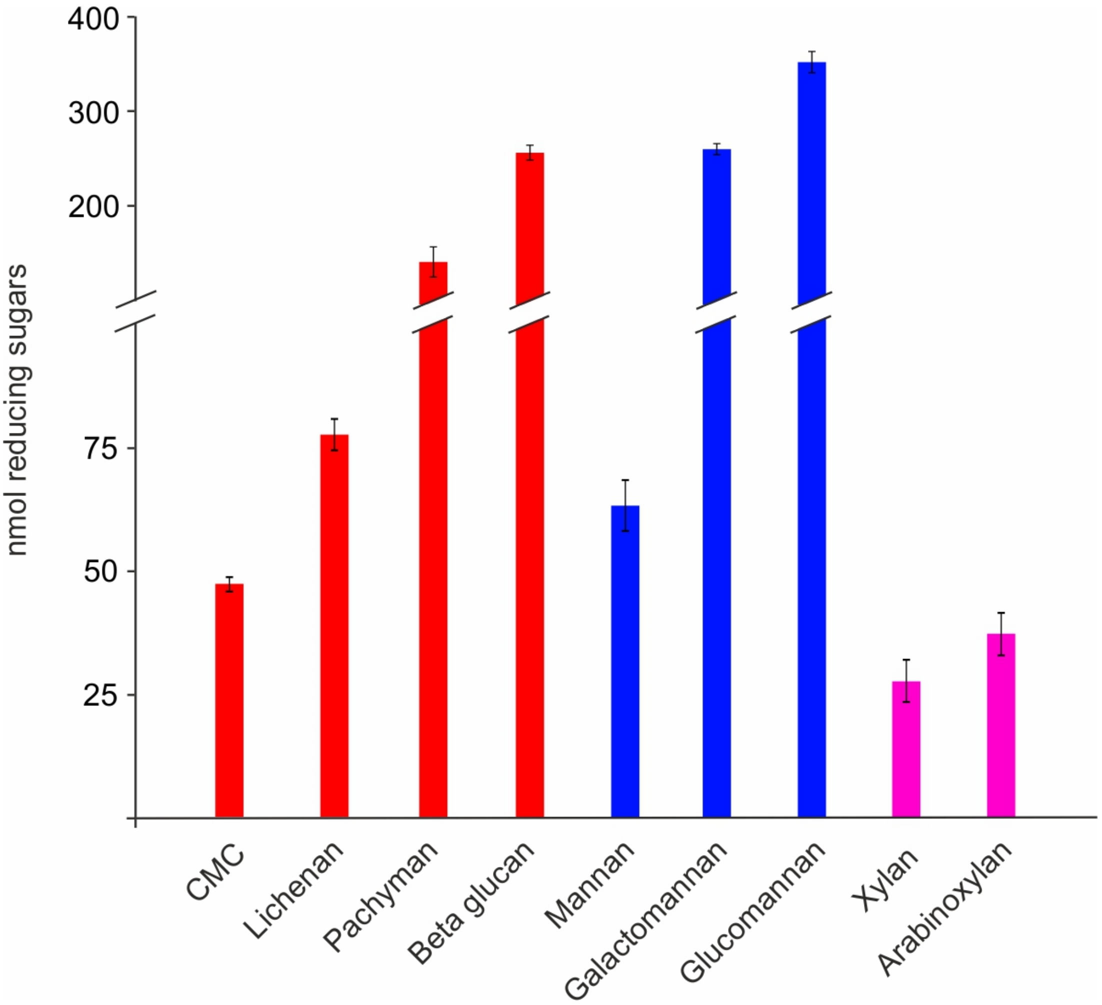

Supplementary Figure 2 | Activity of the soluble extract of *Thermobia*`s crop on a panel of polysaccharides, determined with the dinitrosalicylic acid assay (DNS).

Fifty µL reactions were performed in triplicate in a 96-well plate at 28 °C at 320 rpm using 2.8 µg of protein and 2 mg mL^{-1} substrate in 50 mM sodium phosphate buffer pH 6. After 3 hours, reactions were stopped by adding 100 µL DNS reagent and heating for 10 min at 100 °C, and then absorbance at 540 nm was measured with a microplate reader. All values were blanked against non-enzyme control. Bars indicate means (error bars: standard deviations of three replicates).

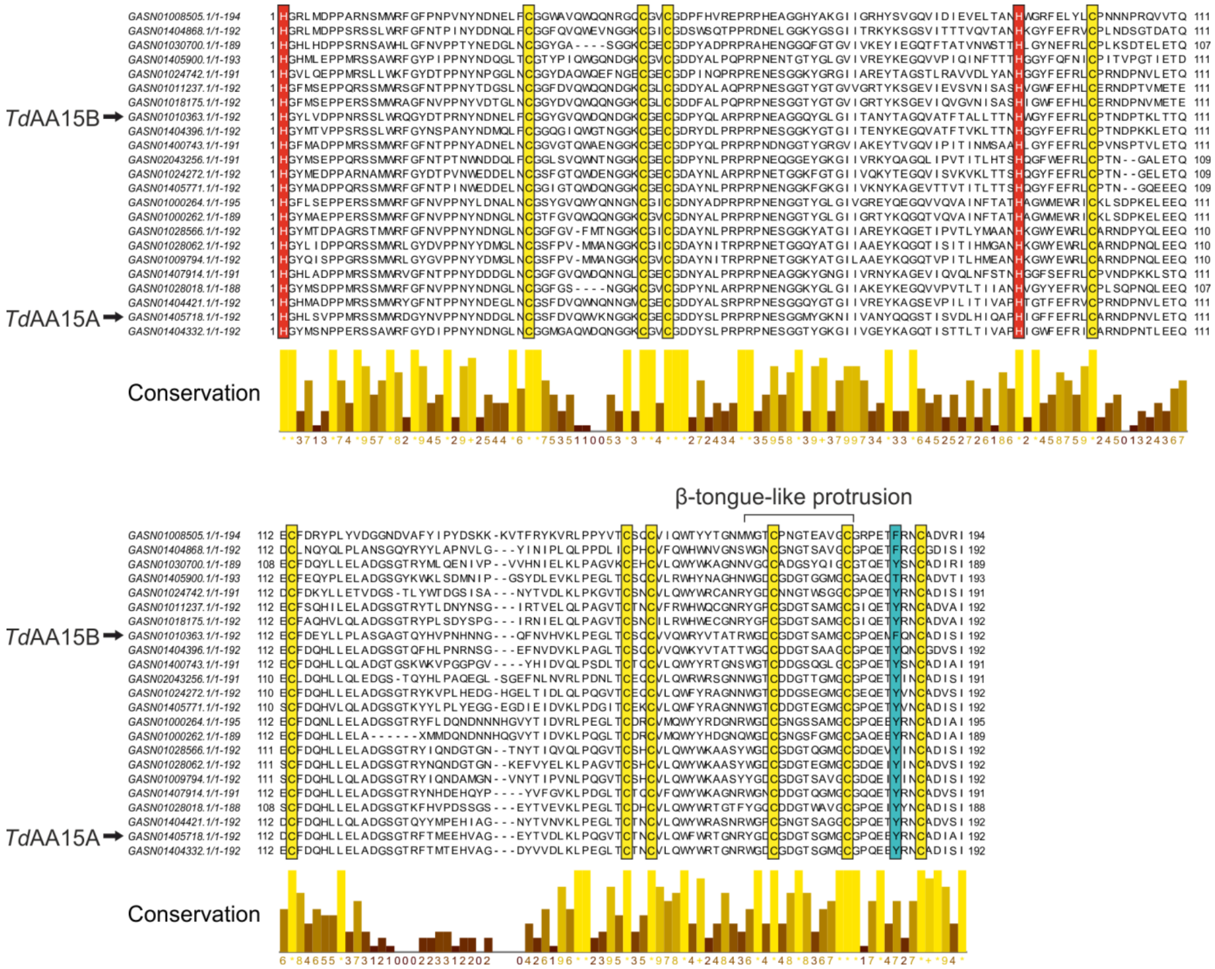

Supplementary Figure 3 | Protein sequence alignment of mature LPMOs (without N-terminal signal peptide) found in the transcriptome of *T. domestica*.

Twenty-three full length LPMO catalytic domains were identified. The conserved histidines involved in copper coordination and the conserved cysteines involved in disulfide bonds are highlighted with red and yellow boxes, respectively. The predicted axial residue is highlighted in blue. Proposed names for the two LPMOs characterized in this study are shown on the left-hand side of the figure. The amino acids involved in the formation of the β-tongue-like protrusion (identified from the X-ray structure of *Td*AA15A) are also indicated.

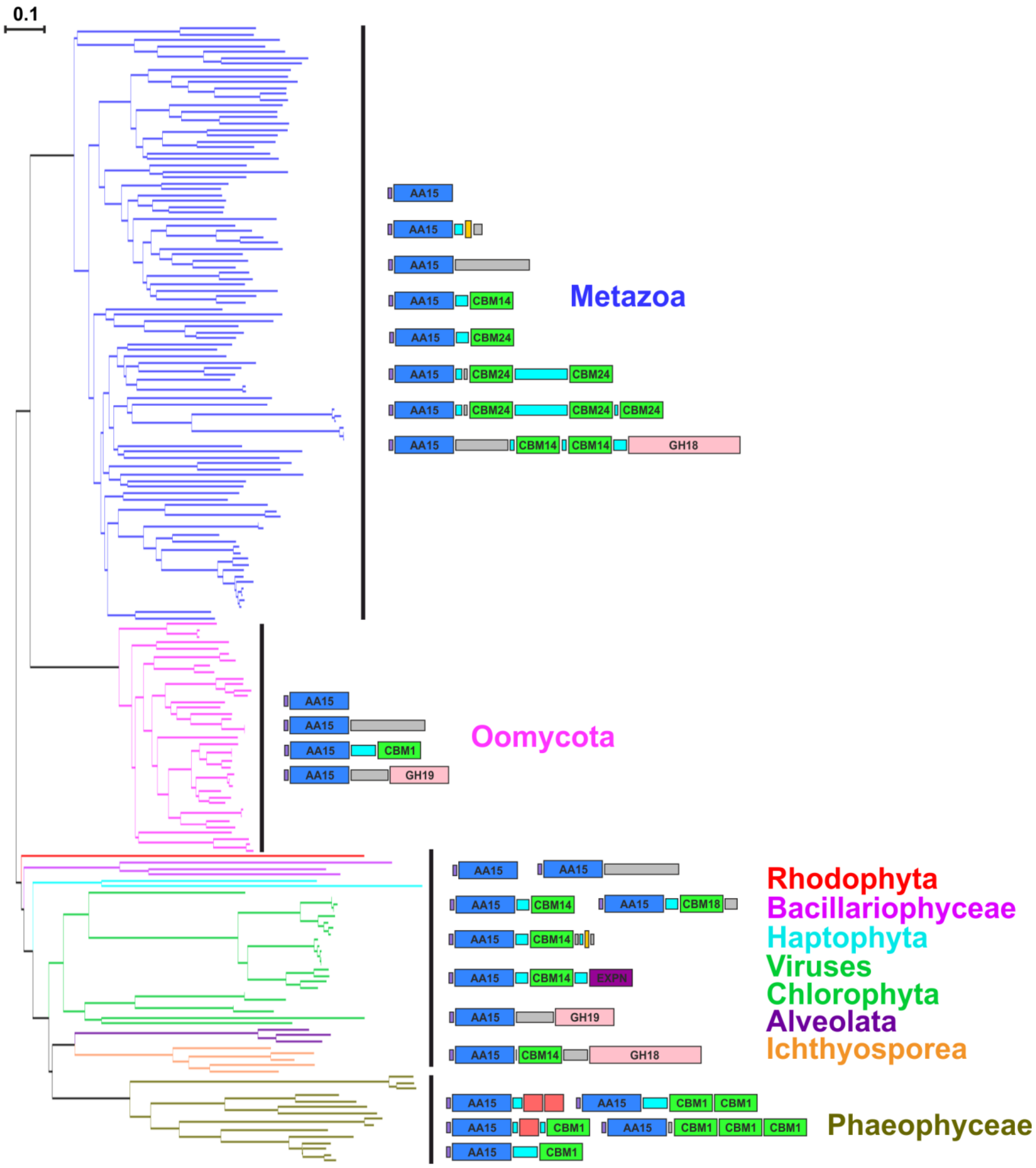

Supplementary Figure 4 | Phylogenetic tree and functional domains of selected members of the AA15 family across Taxa.

To avoid interference from the presence or absence of additional modules, the signal peptides and C-terminal extensions (see below) were removed. The resulting amino acid sequences corresponding to the catalytic domain were aligned using Muscle and built into a phylogenetic tree using the neighbor-joining method. The sequences on the tree were found to segregate according to taxonomy. While *Thermobia*`s sequences only correspond to the

LPMO catalytic domain, about a third of the members of the family harbor a C-terminal extension. Most of these C-terminal extensions could be identified as different carbohydrate-binding domain (CBM) families. Sequences from Phaeophyceae (*Ectocarpus siliculosus*) carry CBM1s, known to bind cellulose. By contrast CBM14s (known to bind chitin) were found in Chlorophyta (and viruses thereof) while family CBM18 (also chitin-binding) was found in Bacillariophyceae. Metazoa divided into two groups, with crustacean, mollusc and insect LPMOs occasionally appended to distant relatives of family CBM24 (too distant to propose a likely carbohydrate target) while all enzymes from *Oikopleura dioica* (a tunicate) carry CBM14 domains. Some viral AA15s were also found to be fused to a putative expansin domain (EXPN). Regions that did not contain the LPMO or putative CBMs are colored in red (domains/regions of unknown function, conserved in other CAZys), purple (signal peptide), cyan (P/T/S-rich linker peptide), yellow (transmembrane segment) and grey (unknown).

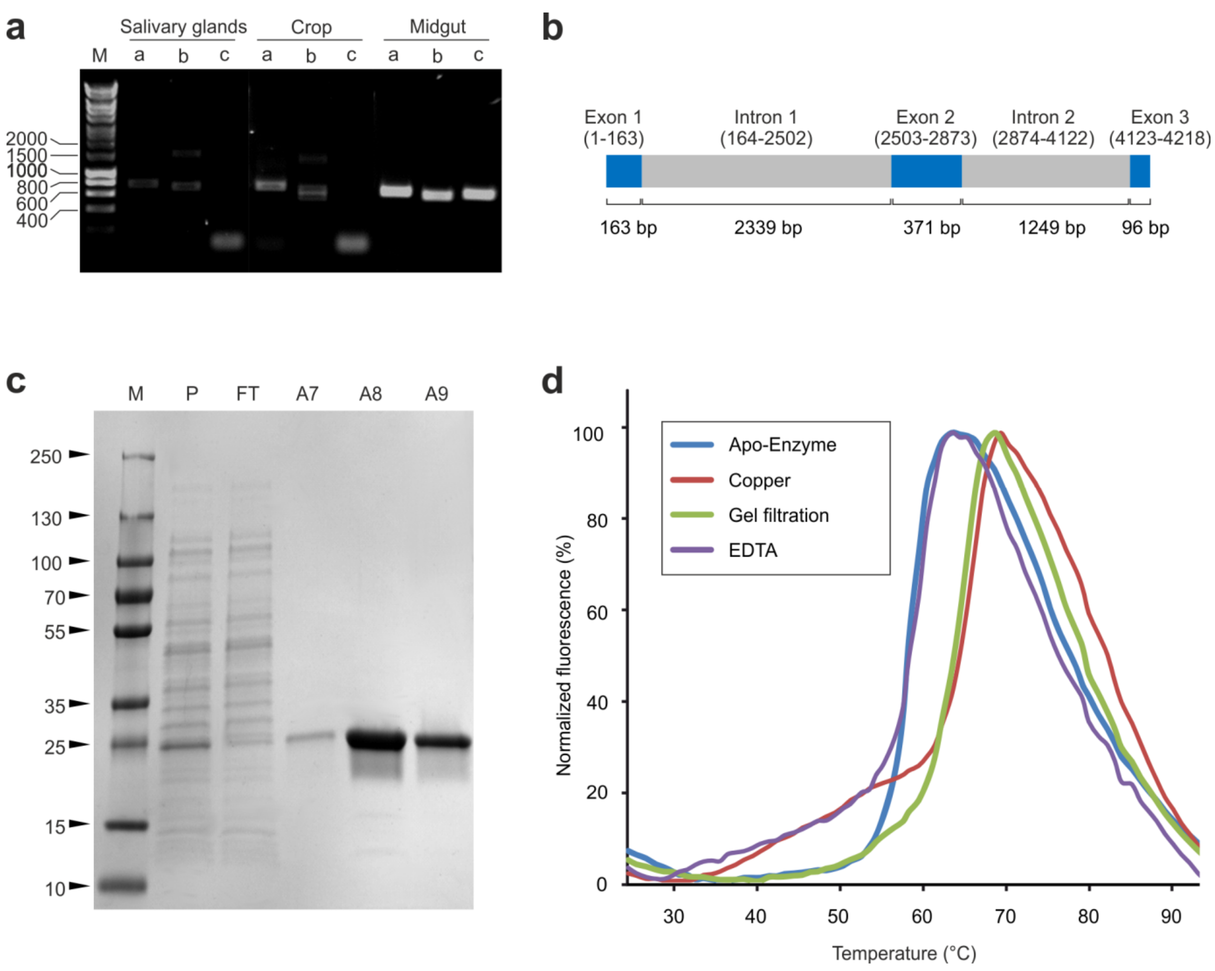

Supplementary Figure 5 | Isolation of LPMO coding sequences from *T. domestica* and heterologous expression of *Td*AA15A in *E. coli*. (**a**) RT-PCR carried out with cDNA template generated from equal amount of RNA extracted from pooled samples of salivary glands, crop and midgut of adult *T. domestica* grown on microcrystalline cellulose. Oligonucleotide primers were designed for LPMO cDNA sequences *GASN01405718.1* (corresponding to *Td*AA15A, lane a), *GASN01404332.1* (lane b) and *GASN01404396.1* (lane c). M = DNA marker. (**b**) Full gene structure of LPMO *GASN01030700.1*, showing the presence of three exons and two introns. The sequence was amplified from genomic DNA extracted from the legs of several specimens, cloned into a plasmid and sequenced *via* primer walking. The full gene sequence can be found in Supplementary Notes. (**c**) SDS-PAGE analysis of periplasmic extract of *E. coli* (P), flow through after passing the periplasmic extract into a 5 mL strep-tag affinity chromatography column (FT), fractions of the protein peak eluted with 2.5 mM desthiobiotin (A7, A8, A9). M: protein marker. Numbers on the left indicate the molecular weight in kDa. (**d**) Melting curves of the recombinant LMPO after strep-tag affinity chromatography (“Apo-Enzyme”, T_m 58.5 °C), after metal loading with 5-

fold molar excess of $CuSO_4$ ("Copper", T_m 64 °C), after size-exclusion chromatography ("Gel filtration", T_m 64 °C) and after de-metallation with 10 mM EDTA ("EDTA", T_m 58.6 °C). A strong increase in thermal stability was observed upon addition of copper and was retained after gel filtration, indicating tight specific binding and protein-fold rearrangement. Such increase was completely reversed by the metal chelator EDTA.

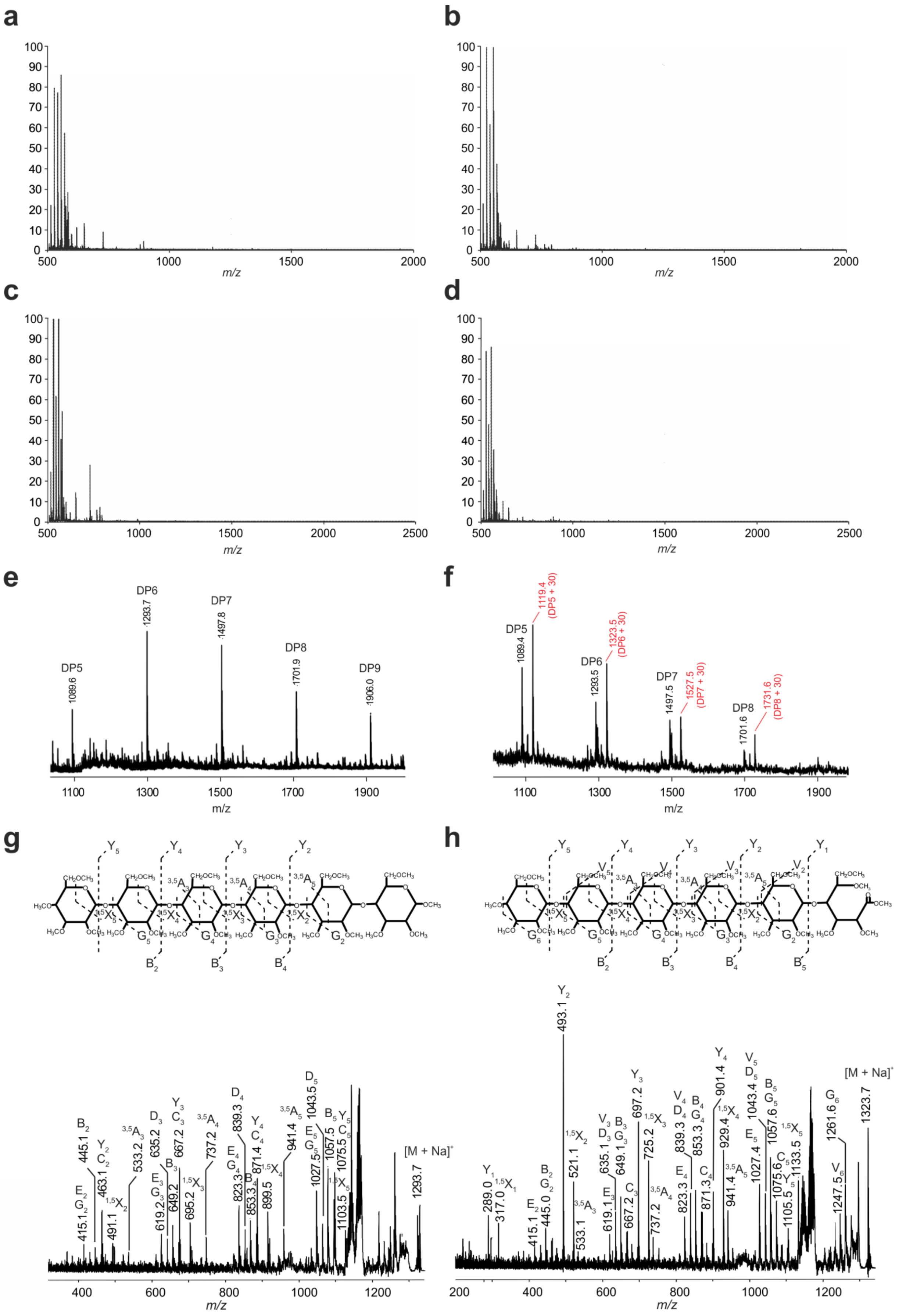

a
b
c
d
m/z
e
DP5
1089.6
DP6
1293.7
DP7
1497.8
DP8
1701.9
DP9
1906.0
f
DP5
1089.4
1119.4 (DP5 + 30)
DP6
1293.5
1323.5 (DP6 + 30)
DP7
1497.5
1527.5 (DP7 + 30)
DP8
1701.6
1731.6 (DP8 + 30)
g
h
[M + Na]+
1293.7
1323.7

Supplementary Figure 6 | MALDI-TOF MS of negative control assays for *Td*AA15A and MALDI-TOF/TOF MS/MS of permethylated products generated by *Td*AA15A when incubated with PASC and electron donor.

(**a-d**) MALDI-TOF MS analysis of *in vitro* negative control activity assays with purified *Td*AA15A, under the same experimental conditions as in Figure 3a, b. The panels show spectra of products obtained after incubation of 4 mg mL^{-1} microcrystalline cellulose (**a, b**) or β-chitin (**c, d**), either with 4 mM gallic acid **(a, c)** or 2 μM *Td*AA15A (**b, d**) alone. The spectra do not show detectable amounts of native or oxidized cello-oligosaccharides. In **a-b** and **c-d**, 100% relative intensity represents 0.9 x 10^4 and 1.0 x 10^4 arbitrary units (a.u.), respectively. (**e-h**) Mass spectrometry analysis of permethylated products of LPMO cleavage of PASC. *Td*AA15A and PASC were incubated in the absence (**e**) or presence (**f**) of 4 mM gallic acid before permethylation and analysis by MALDI-TOF MS. With the LPMO and reducing agent, a series of additional products 30 Da greater than the cello-oligosaccharides was detected, consistent with an aldonic acid modification (C1-oxidation). (**g, h**) MALDI-TOF/TOF MS/MS of permethylated oligosaccharides. Diagrams show the origin of observed fragmentation ions. The nomenclature is according to Domon & Costello (1988)[56]. Fragmentation of the non-oxidized cellohexaose $[M+Na]^+$=1294 is shown in **g**, and the oxidized oligosaccharide $[M+Na]^+$=1324 in **h**. The series of ^{1,5}X and Y ions comprising the reducing end of the oxidized species are 30 Da larger than those of the non-oxidized species, whereas the B ions comprising the non-reducing end are identical. Consequently, the oxidation is found on the reducing end residue.

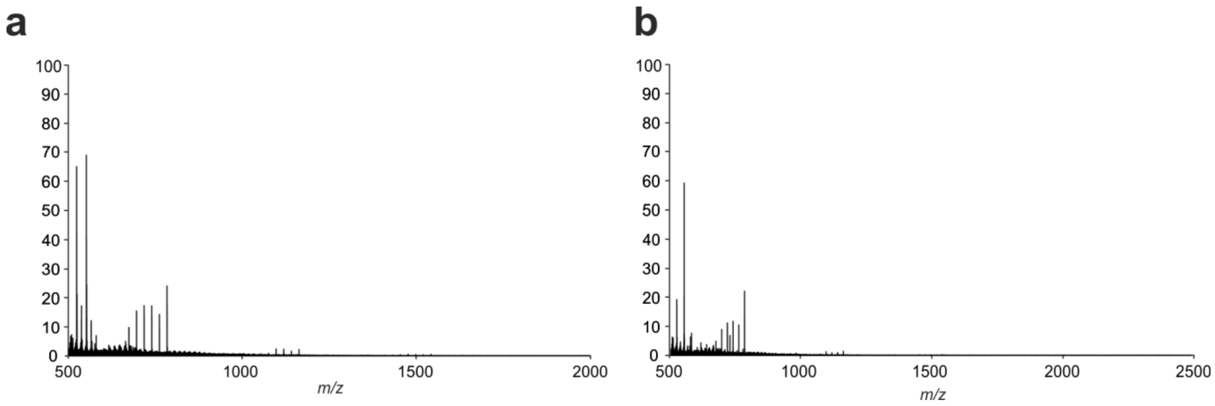

Supplementary Figure 7 | MALDI-TOF MS of activity assays with *Td*AA15A in presence of EDTA.

MALDI-TOF MS analysis of *in vitro* negative control activity assays with purified *Td*AA15A, under the same experimental conditions as in Figure 3a, b. The panels show spectra of products obtained after incubation of 4 mg mL^{-1} microcrystalline cellulose (**a**) or β-chitin (**b**) with 2 μM *Td*AA15A, 4 mM gallic acid and 10 mM EDTA. The spectra do not show detectable amounts of native or oxidized cello-oligosaccharides. In **a** and **b**, 100% relative intensity represents 0.9 x 10^4 and 1.0 x 10^4 arbitrary units (a.u.), respectively.

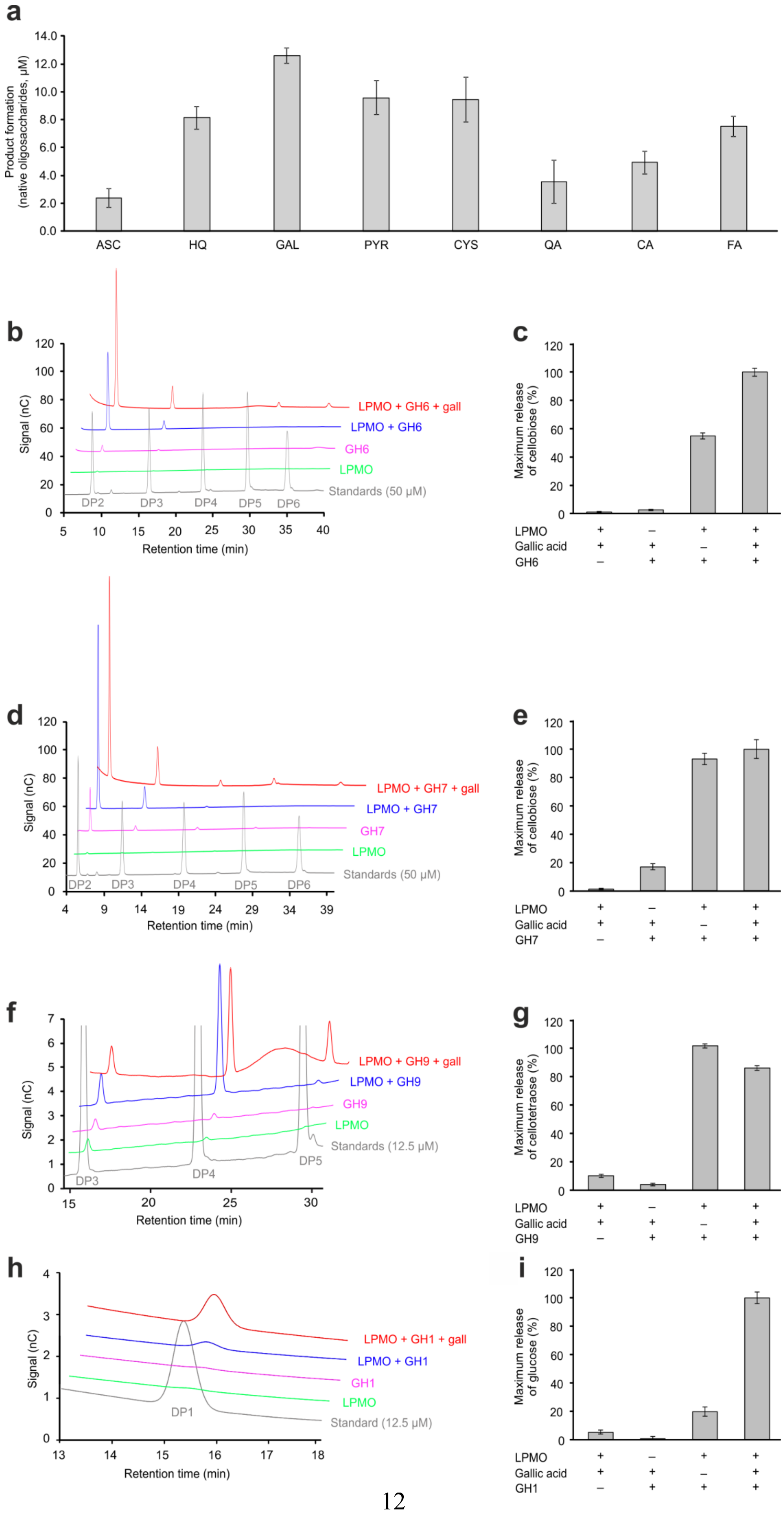
a
Product formation (native oligosaccharides, µM)
14.0
12.0
10.0
8.0
6.0
4.0
2.0
0.0
ASC
HQ
GAL
PYR
CYS
QA
CA
FA
b
Signal (nC)
120
100
80
60
40
20
0
5
10
15
20
25
30
35
40
Retention time (min)
LPMO + GH6 + gall
LPMO + GH6
GH6
LPMO
Standards (50 µM)
DP2
DP3
DP4
DP5
DP6
c
Maximum release of cellobiose (%)
120
100
80
60
40
20
0
LPMO + – + +
Gallic acid + + – +
GH6 – + + +
d
Signal (nC)
120
100
80
60
40
20
0
4
9
14
19
24
29
34
39
Retention time (min)
LPMO + GH7 + gall
LPMO + GH7
GH7
LPMO
Standards (50 µM)
DP2
DP3
DP4
DP5
DP6
e
Maximum release of cellobiose (%)
120
100
80
60
40
20
0
LPMO + – + +
Gallic acid + + – +
GH7 – + + +
f
Signal (nC)
7
6
5
4
3
2
1
0
15
20
25
30
Retention time (min)
LPMO + GH9 + gall
LPMO + GH9
GH9
LPMO
Standards (12.5 µM)
DP3
DP4
DP5
g
Maximum release of cellotetraose (%)
120
100
80
60
40
20
0
LPMO + – + +
Gallic acid + + – +
GH9 – + + +
h
Signal (nC)
4
3
2
1
0
13
14
15
16
17
18
Retention time (min)
LPMO + GH1 + gall
LPMO + GH1
GH1
LPMO
Standard (12.5 µM)
DP1
i
Maximum release of glucose (%)
120
100
80
60
40
20
0
LPMO + – + +
Gallic acid + + – +
GH1 – + + +

Supplementary Figure 8 | Identification of the best electron donor and synergy experiments with purified *Td*AA15A using PASC as substrate.

(**a**) Native oligosaccharides with a degree of polymerization from 2 to 6 were released from 1 mg mL^{-1} PASC by the purified LPMO during 24-hour experiments in the presence of 1 mM reductant and quantified *via* HPAEC. ASC: ascorbic acid. HQ: hydroquinone. GAL: gallic acid. PYR: pyrogallol. CYS: cysteine. QA: quinic acid. CA: coumaric acid. FA: ferulic acid. All experiments were done in triplicates. All values shown here have been blanked against reactions containing the LPMO without any electron donors. Bars indicate means (error bars: standard deviations of three replicates). It was decided to quantify the native oligomers, instead of C1-oxidized products, because they are more stable and ensure a reliable comparison between conversion experiments, as previously reported[24]. (**b-i**) 100 µL reactions containing 1 mg mL^{-1} substrate, 2 µM LPMO, 1 mM gallic acid and different amounts of commercial glycoside hydrolases (0.8 mU GH6 (**b, c**), 5.4 mU GH7 (**d, e**), 10 µg GH9 (**f, g**) and 4 mU GH1 (**h, i**)) were carried out at 28 °C for 3 hours at 600 rpm. Oligosaccharides were quantified by HPAEC. The LPMO boosted the activity of all GHs tested, and the synergy was further enhanced upon addition of gallic acid. The main products released were cellobiose (GH6, GH7), cellotetraose (GH9) and glucose (GH1). The chromatograms of all boosting experiments are staggered in order to avoid overlapping of the same peak from different samples. Bars indicate means (error bars: standard deviations of three replicates). Two different HPAEC methods were used for **b-g** ("oligosaccharide method") and **h-i** ("monosaccharide method"), respectively (see Methods for more details). We routinely experienced a slight shift of the retention time for the same peak (for example, cellobiose) between different sample batches (**b, d** and **f**) which, however, did not interfere with the analysis. The identity and concentration of the products was determined by analysis of commercial standards.

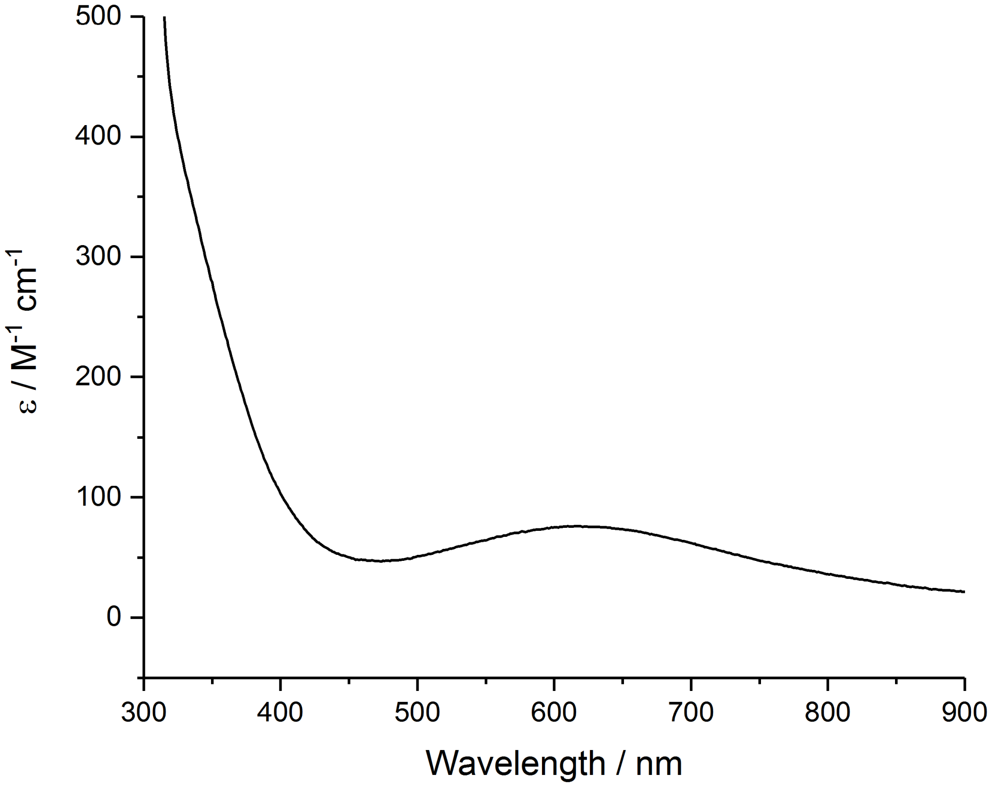

Supplementary Figure 9 | UV/vis spectrum of *Td*AA15A. Spectrum was collected with 1 mM protein in 20 mM sodium phosphate buffer pH 7.

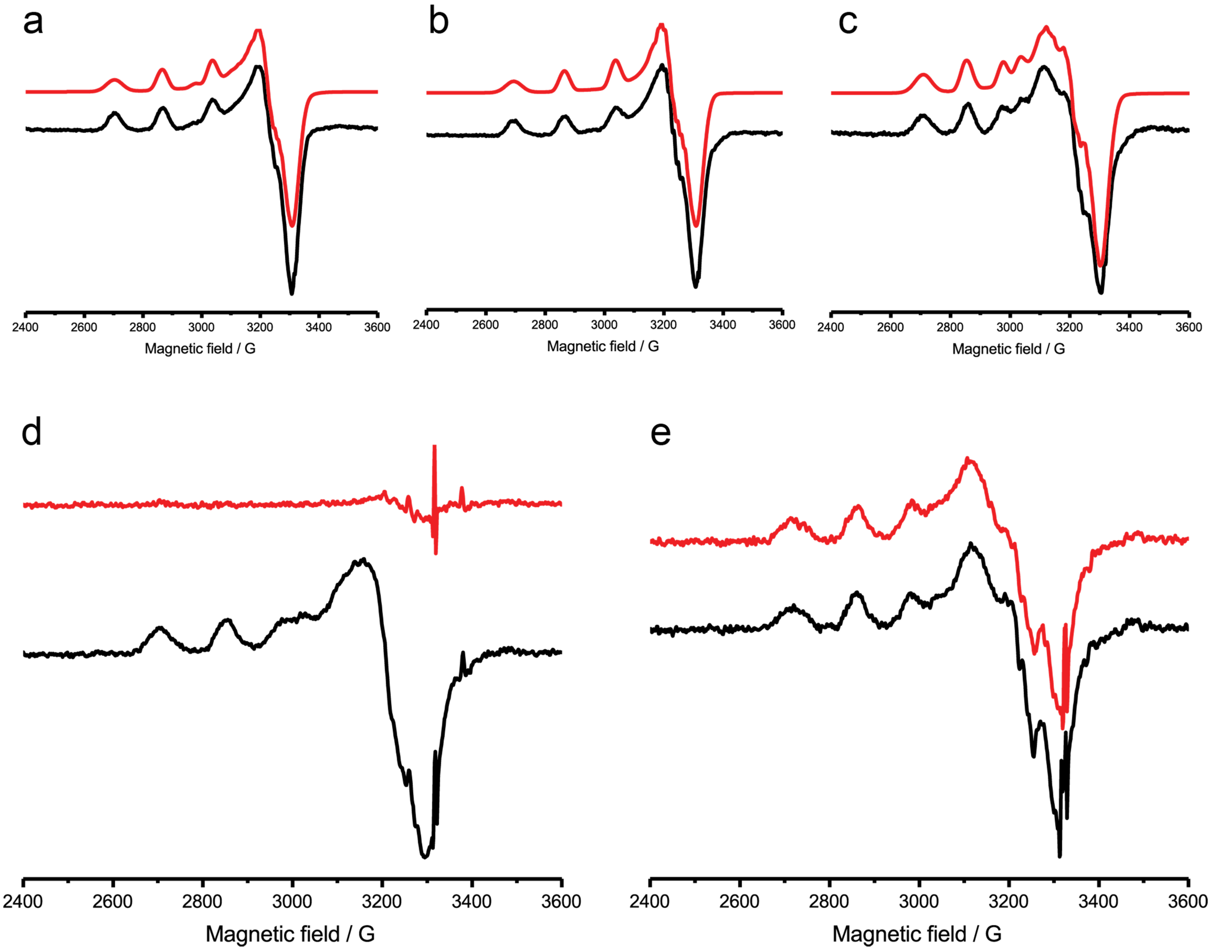

Supplementary Figure 10 | Continuous wave X-band EPR spectra (9.3 GHz, 160 K) of *Td*AA15A. EPR spectra of 0.3 mM *Td*AA15A in a mixed buffer composed by sodium acetate, MES, HEPES and TRIS (10 mM each) at pH 6 (**a**), pH 8.5 (**b**) or pH 6 with 10% glycerol added (**c**), with simulations shown in red. Simulations were obtained with 85% of species 1 and 15% of species 2 in (**a**), 100% of species 1 in (**b**) and 45% of species 1 and 55% of species 2 in (**c**). See Supplementary Table 3 for details of spin Hamiltonian parameters. (**d**) EPR spectrum of 0.2 mM *Td*AA15A in 20 mM sodium phosphate buffer pH 6 recorded before (black) or 10 min after addition of 100 equivalents of the reductant sodium ascorbate (red). (**e**) EPR spectrum of 0.2 mM *Td*AA15A in 20 mM sodium phosphate buffer pH 6 with 10% glycerol recorded before (black) or 10 min after addition of 20 equivalents of the oxidant potassium ferricyanide (red). The signal at ~3320 G in (**d**) and (**e**) is due to the EPR tube.

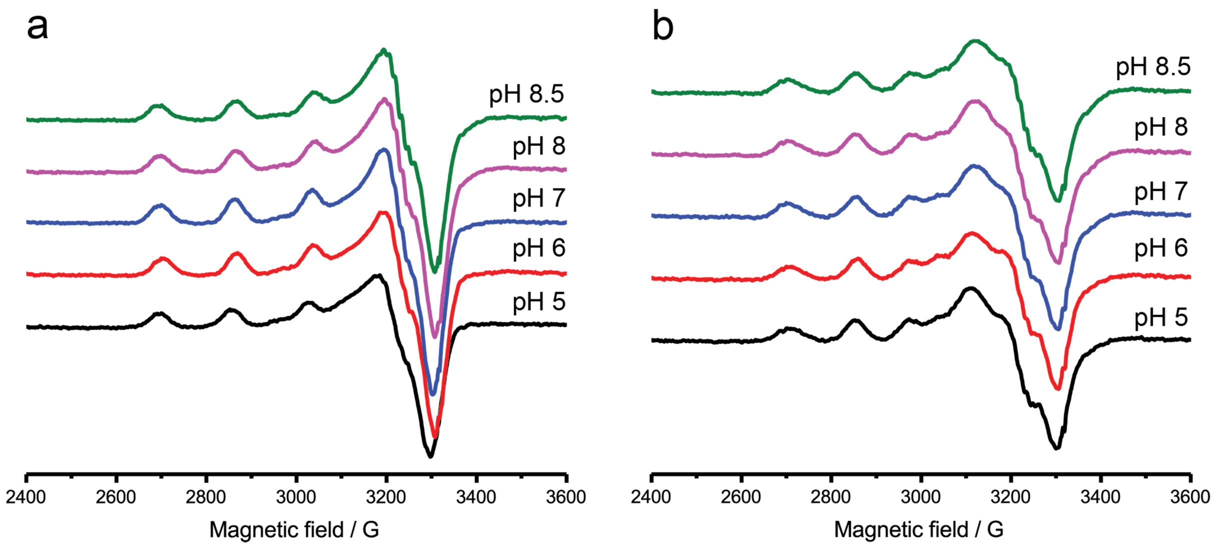

Supplementary Figure 11 | Continuous wave X-band EPR pH titration spectra (9.3 GHz, 160 K) of *Td*AA15A. EPR spectra of 0.3 mM *Td*AA15A in a mixed buffer composed by sodium acetate, MES, HEPES and TRIS (10 mM each) without (**a**) and with (**b**) 10% glycerol. Spectra recorded every 0.5 pH units between pH 5 and pH 8.5. For clarity, only a selection of spectra is presented; full data are available through Research Data York (DOI: 10.15124/bd09e86b-9d92-4802-9337-18b138e7abb7).

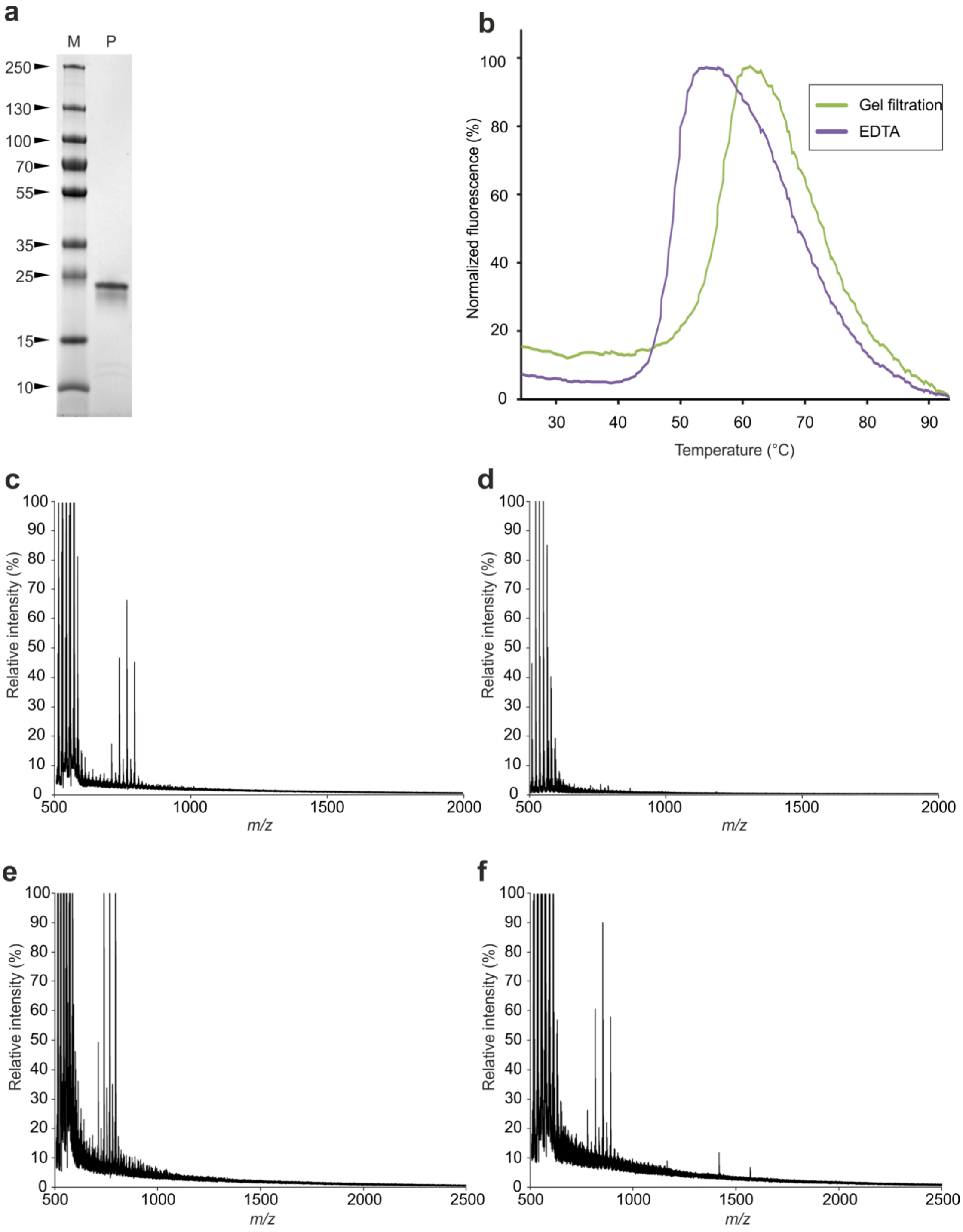

Supplementary Figure 12 | Purification of recombinant *Td*AA15B and negative control activity assays with chitin.

(**a**) SDS-PAGE analysis of *Td*AA15B after purification with a 5 mL strep-tag affinity chromatography column followed by size-exclusion chromatography. P: protein. M: protein marker. Numbers on the left indicate the molecular weight in kDa. (**b**) Melting curves Cu-loaded *Td*AA15B after size-exclusion chromatography ("Gel filtration", T_m 56.7 °C) and after de-metallation with 10 mM EDTA ("EDTA", T_m 49.2 °C). (**c-f**) MALDI-TOF MS analysis of *in vitro* negative control activity assays with purified *Td*AA15B, under the same

conditions as in Fig. 5 b, c. The panels show spectra of products obtained after incubation of 4 mg mL^{-1} α-chitin (**c, d**) or β-chitin (**e, f**), either with 4 mM gallic acid (**c, e**) or 2 μM *Td*AA15B (**d, f**) alone. The spectra do not show detectable amounts of native or oxidized cello-oligosaccharides. In **c-d** and **e-f**, 100% relative intensity represents 2.9 x 10^4 and 1.3 x 10^4 arbitrary units (a.u.), respectively.

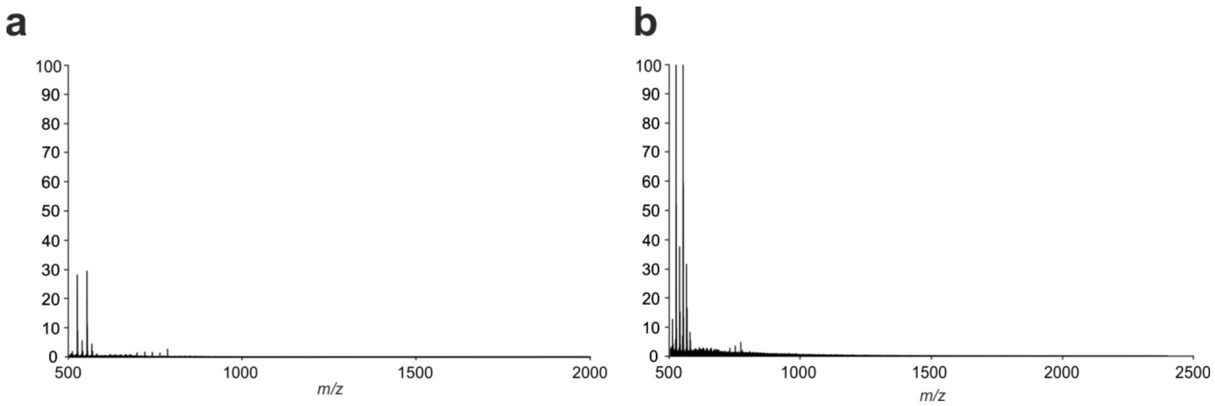

Supplementary Figure 13 | MALDI-TOF MS of activity assays with *Td*AA15B in the presence of EDTA.

MALDI-TOF MS analysis of *in vitro* negative control activity assays with purified *Td*AA15B, under the same experimental conditions as in Figure 5b, c. The panels show spectra of products obtained after incubation of 4 mg mL^{-1} α-chitin (**a**) and β-chitin (**b**) with 2 μM *Td*AA15B, 4 mM gallic acid and 10 mM EDTA. The spectra do not show detectable amounts of native or oxidized cello-oligosaccharides. In **a** and **b**, 100% relative intensity represents 2.9 x 10^4 and 1.3 x 10^4 arbitrary units (a.u.), respectively.

Supplementary Table 1 | Gene expression analysis of LPMO sequences in *T. domestica*.

Contig	Number of mapped reads	TPM
GASN01405718.1 = *Td*AA15A	49220	2040
GASN01030700.1	41936	1363
GASN01405900.1	19092	777
GASN02043256.1	20908	839
GASN01018175.1	19326	657
GASN01000262.1	11244	415
GASN01407914.1	10437	345
GASN01404332.1	5175	243
GASN01000264.1	2942	145
GASN01024742.1	2475	129
GASN01028566.1	1285	78
GASN01400743.1	1198	73
GASN01404421.1	1491	69
GASN01009794.1	1149	66
GASN01404396.1	728	34
GASN01028018.1	410	22
GASN01011237.1	552	22
GASN01028062.1	300	18
GASN01024272.1	303	13
GASN01405771.1	136	6
GASN01404868.1	74	3
GASN01010363.1 = *Td*AA15B	74	1
GASN01008505.1	41	1

Raw reads were retrieved from NCBI (accession: SRR921648) and mapped onto the published transcriptome of *Thermobia* to determine normalized expression values (TPM = Transcripts Per kilobase Million) using Salmon (part of the Galaxy toolshed)[53].

Supplementary Table 2 | Data collection, phasing and refinement statistics of *Td*AA15A.

	Cu^{2+} SAD - BLEND	Cu(I) (5MSZ)
**Data collection**		
Space group	$P22_12_1$	$P22_12_1$
Cell dimensions		
a , *b* , *c* (Å)	47.0, 56.9, 66.9	47.0, 56.9, 66.7
α, β, γ (°)	90.0	90.0
Wavelength (Å)	0.966	0.966
Resolution (Å)	66.87–2.00 (2.05–2.00)	56.87-1.10 (1.12-1.10)
R_{merge}	0.12 (0.15)	0.07 (0.66)
$I / \sigma I$	44.3 (31.8)	8.0 (1.6)
Completeness (%)	100.0 (100.0)	99.6 (99.9)
Redundancy	54.1 (57.0)	3.8 (3.8)
Half-set correlation CC(1/2)	0.998 (0.998)	0.996 (0.637)
$R_{p.i.m.}$	0.022 (0.028)	0.054 (0.580)
**Refinement**		
Resolution (Å)		56.87-1.10
No. reflections (Work/Free)		72,815/3694
R_{work} / R_{free}		0.13/0.15
No. atoms		
Protein		2,967
Ligand/ion		19
Water		209
B -factors (Å^2)		
Protein		15
Ligand/ion		35
Water		25
R.m.s deviations		
Bond lengths (Å)		0.012
Bond angles (°)		1.55

Supplementary Table 3 | EPR analysis of *Td*AA15A

		Species 1	Species 2
	g_x	2.044	2.017
**g values**	g_y	2.071	2.115
	g_z	2.254	2.283
	A_x	47	15
**A_{Cu} (MHz)**	A_y	60	65
	A_z	525	407
**SHF A_N (MHz)**		18, 31, 37	30-40
**A_{cu} strains (MHz)**		57, 65, 100	70, 75, 100
**Line widths**		0.3, 0.3	0.3, 0.3

EPR spin Hamiltonian parameters from simulations of cw X band spectra for *Td*AA15A. The EPR parameters were derived by simultaneous fitting of the spectra at pH 6, pH 8.5 and pH 6 in the presence of 10% glycerol obtained during the EPR pH titrations (Supplementary Figure 10). Parallel values are determined accurately. Accurate determination of perpendicular values and superhyperfine coupling constants was not possible due to second order nature of the spectrum in this region.

Supplementary Table 4 | List of chitin-related genes co-expressed with *Td*AA15A in *Drosophila* across developmental stages (from embryo to adult).

Gene	Correlation (%)	Main molecular function	Main biological processes
Cpr65Ec	88.65	structural constituent of chitin-based larval cuticle	chitin-based cuticle development
Edg78E	84.13	structural constituent of chitin-based larval cuticle	chitin-based cuticle development
CG13676	83.51	chitin binding	chitin metabolic process
Cpr51A	82.59	structural constituent of chitin-based larval cuticle	chitin-based cuticle development
reb	82.02	unknown	chitin biosynthetic process
tfc	81.77	carbohydrate binding	adult chitin-containing cuticle pigmentation
Cpr49Ag	80.69	structural constituent of chitin-based larval cuticle	chitin-based cuticle development
Edg91	80.26	structural constituent of pupal chitin-based cuticle	chitin-based cuticle development
CG8927	79.65	structural constituent of cuticle	chitin-based cuticle development
TwdlE	78.76	structural constituent of chitin-based cuticle	chitin-based cuticle development
obst-B	78.37	chitin binding	chitin metabolic process
pot	78.02	molecular_function	chitin-based embryonic cuticle biosynthetic process
CG10359	77.42	G-protein coupled receptor binding<br>chitin binding	G-protein coupled receptor signaling pathway
Cht6	77.23	chitinase activity	chitin catabolic process
kkv	76.92	chitin synthase activity	chitin biosynthetic process<br>trachea morphogenesis
pio	76.64	molecular_function	chitin-based embryonic cuticle biosynthetic process<br>open tracheal system development
obst-A	76.48	chitin binding	chitin metabolic process<br>regulation of tube size, open tracheal system
Cda4	75.99	hydrolase activity, acting on carbon-nitrogen (but not peptide) bonds	chitin metabolic process
Gfat1	75.78	glutamine-fructose-6-phosphate transaminase (isomerizing) activity	carbohydrate derivative biosynthetic process
Cht5	75.44	chitinase activity	chitin catabolic process
Lcp65Ad	75.36	structural constituent of chitin-based larval cuticle	chitin-based cuticle development
Cpr64Ad	75.06	structural constituent of chitin-based larval cuticle	chitin-based cuticle development
Cpr97Ea	74.10	structural constituent of chitin-based larval cuticle<br>structural constituent of cuticle	chitin-based cuticle development
knk	73.16	unknown	chitin biosynthetic process<br>trachea morphogenesis
Cpr56F	72.98	structural constituent of chitin-based larval cuticle	chitin-based cuticle development
Cht7	71.43	chitinase activity	chitin catabolic process

The table shows the chitin-related genes found among the top 100 sequences with the highest expression correlation with *Td*AA15A during embryogenesis, larval stage, pupal stage and adult stage. modENCODE developmental RNA-Seq data were retrieved from FlyBase using the RNA-Seq Expression Similarity Search tool.

Supplementary Table 5 | Phenotypes of *T. castaneum* induced by RNAi knockdown of AA15 coding genes (Data: iBeetle).

Gene target of RNAi	Phenotype for "Metamorphosis and survival"
TC016344	Lethalities 11 days after larval injection: 40.0% (includes death as larva, prepupa, pupa)
	Lethalities 22 days after larval injection: 40.0% (includes death as larva, prepupa, pupa, adult)
TC016345	Lethality 11 days after pupal injection: 70.0% (includes death as pupa, adult)
TC016348	Lethality 11 days after pupal injection: 60.0% (includes death as pupa, adult)
TC016349	Thorax not present
	Abdomen not present
	Percentage of animals affected/eggs on slide: more than 80%
TC016350	Lethality 11 days after pupal injection: 30.0% (includes death as pupa, adult)
	Embryo/egg without visible larval cuticle in cuticle preparation - percentage of animals/eggs on slide: more than 80%
TC002263	Embryo/egg without visible larval cuticle in cuticle preparation - percentage of animals/eggs on slide: more than 80%

Supplementary Table 6 | List of oligonucleotide primers.

Oligonucleotide name	Oligonucleotide sequence
GASN01405718.1_F	TGGTAAAGTTATCGCAGCAGATC
GASN01405718.1_R	AAGGAGGAAGTAGCTGCCTAC
GASN01404332.1_F	TTCACAATGGCTCGTGTAGC
GASN01404332.1_R	TCGTTGAAAATGTGTGAGATACCTG
GASN01404396.1_F	AGCAAGTAAAAGAACTAAA
GASN01404396.1_R	AGTAGTTGTGTCTTCTTTACTGTCCTG
GASN01405718.1_InFus_F	CAGCCGGCGATGGCCCATGGACACTTGTCCGTTCC
GASN01405718.1_Strep_R	CTTTTCGAACTGCGGGTGGCTCCAAGCGATAGCAATATC AGCACAATTTC
GASN01010363.1_InFus_F	CAGCCGGCGATGGCCCATGGATATCTGGTAGATCCGC
GASN01010363.1_Strep_R	CTTTTCGAACTGCGGGTGGCTCCAGGCAATGCTTATATC AGCACAATTC
Strep_R_InFus	AGCAGCCGGATCTCACTTTTCGAACTGCGGGTG

Supplementary Notes

Supplementary Note 1

Full gene coding sequence (corresponding to contig GASN01030700.1) from start to stop codon, amplified from genomic DNA of *T. domestica* (exons are underlined).

ATGGCTCGTCTAATCTTGGCAATCAGCGCTCTCCTCTTGGCCCACCAAGTTGC
AGGCCATGGTCACTTGCACGACCCTCCTTCCAGAAACTCCGCTTGGCATCTG
GGATTCAATGTTCCTCCTACCTACAACGAGGATGGACTTAACTGTGGTGGCT
ATGGAGTGAGTAACACCTTAATATGTTTAAAATCAGCATATTCTTGATCCATC
CTTTTATTAAATCTTTGCAAAGACCCTGAACTATAGGACTTTTCATGTTCAAC
GGTGGGAAGTAGCAGTAAAATAGTAAGTTTGTTAAGTTTGTTCGTAATGTTT
CCAATAAAACAGAGCACGAATTTCTTTGGAAGAAAGGATGGAAAATAATAA
AAAGAAAATAGGAAACTAAAGAATTAAGCAAGAAGTAGAAAAGAAATTGGT
AGTTAGGCATATTCGTACATACGTTCGTATCTTAATTTTTACATCGATAATTC
GAGGAATCTTCATTGGTATATTTAGACGTAATATTAAAAGAATTACTAAAAT
TTTGTAACGGTAGAAAATTATTCAAAGAGGTTTGTACGTAGTAGTGGAGATG
TAGATAAGAAAATGCCATCGTGGTAATGGAAATGCAAGTAAACGAATTTAGT
TTGCATTATAGAGATGAAAGCAGGAGAAATCTTCGTTTACCAATTCTTTTAAC
TTAACACGTAGACTACCATGTGTATGAAATTCTAAACACATGGCTCCCCCGT
ACAGCGCCGTGTGTATGATTTTCCATACACAAACAATATATTGCACTTTTGTC
GCGTTCGTTCTATAGGTTTGTATGCAATTACATACATATGGCGAATAAGTCAA
AATTTAATGTCCAAGCCACGTATGTACAAAACCATACAGAAAGAAAATTAAC
ATTTCTGCGGCTAAAACAAAGATGGCGGCCCGTGGCACACAGGCGATAACG
ATCAACAGATATAGTGGCAGCCAAAGTGTTAAAAAAAATAAATGTGTGAAA
TAAAGTAATCACATTTAAACATAAAAATTCTAACTATCAGAAGACCCCTGAA
ATATCTCATTAATTATAAATTACCTTGAGCTGTGGTATATTTTTTTAAAAAAA
ATATTTTTACATCATATTTTATTTTCGAGTACACAACAGTTAAATACCAGGGT
GTTTTCCATTTGCACAAGGTCACTGTTTAATATAGGTTTAGTTATTGTCCTTG
ACACATCACAAACATAGATATCCTATTAGGGAAAGCATCCAGTCAGCATTAT
ACGTACTTGACTAATGATAAAGTGTTAACAGAATTTGGGGGAAAAGAAGAA
CTATATAAAGTCAATTTAGAAGAAATTGTCATATTAGGGTTGTGATAAAATA

ATAGAAGGTGACTGAGGGAAAGAGAGAAGATTGTACTTGATGCAGGGTATG
AAAGAGGGTAATTCGTAACTCAGAACTAAGAGATTAGATGATCAAGGAAAT
TAGTTGGATGGATAAGATTTCAAACAAAAGTGTGATGAAAATGGTGGACTAT
TTTGAAAAGAGGAGATAGATGATAGGCTATGTTGTGAGACATAGACAAATC
ACGCTGTTAACAACAGAGGGAAGCCAGATAGAACAAGAAAATTAGGAAAAC
CAAAAATGGATCAAAAGATTGCGATCTTGATCAATAATTAGTTTTTGAATCA
GGGGCCACAAAATTTTCAAGCGGTTTCTAAATCAAATTTTCGTATTTTCTAAG
GAATAAGCACATCATACTGAGTTAAGTGATAGTTTGGGTTTGGCTACACTGT
ATGTAGTTCTCCAGATATGAGGCATGCAAGAAAATTTTCGATTGAGATACAT
TGGATCTCTTGATCCAAAAATACTTATAGGAAGCCCACTATTGAAACATACG
GAAAAATTAACACACGCTATGAGGAAAAAGAATACATAGTAGGCTAAGGTA
AGGAAGAATGGATAGATGTATCAAATCAGTCAAAAGATTGCAATATATATAT
ATATTTCTTCGGGAGAATTAAGACTATAGATTCAAACTTGCTAAAGACTAAA
CCCTTAGCAACCATGTTTGAAGACTTCAAAGGGAGTTATTATATGACAGGGA
TATCTTGAAAAGGGTCACGACACCAACAGCCTCATTGAGAGTTTAGGCGGAA
TACTTTTAAACTAGTTAGCAACTAAAAGTTGTATGCAAACTTTATAATAATTT
TTCTTTTAAATAATGGCTGCACCATAAAGGAGAACAATATTAGAGATTAGAA
CCATCGAGAAACTGTAAAAGTAGATACAGTTAAGAGAATTTAGGAGGATTA
GTCATATGTCTTATATTACGAAGGATCGGCACTATGCATAATGGAACGCGTG
GCTGGAACAGTTATGCATTAATTGAGAAACGCACTCATTTCTGATTATCGCTC
ATTCCTCCCCAAAACTCTCTATTAGGAGTGTTGTTTTCTGTTTTTCTTAATAGG
ACAAAATCTACTAAGCACGTTTTACTAAATGAAAACTTCGTTTTTCCTTTCAG
GCAAGTGGTGGCAAATGTGGAGAATGTGGAGATCCTTATGCTGATCCTCGTC
CCCGTGCTCACGAAAATGGTGGCCAATTCGGCACTGGCGTCATCGTCAAGGA
GTACATAGAGGGTCAAACCTTCACCGCAACTGTGAACTGGTCCACCACTCAT
TTGGGATACAACGAGTTCCGTCTGTGCCCACTCAAGAGTGACACTGAACTCG
AGACCCAAGAGTGCTTCGACCAATATCTTCTGGAATTGGCTGATGGCTCTGG
AACCCGCTACATGCTGCAGGAAAACATCGTTCCTGTCGTTCACAACATCGAG
CTCAAACTTCCAGCTGGTGTCAAATGTGAACACTGTGTCCTCCAATGGTACTG
GAAGGCCGGTATGTGTATTTTTAGTATTTTTCATCATCATGCATTGAAACTTT
AATACTGAAATGAGTCATATTTATCGTAATATTTTAAATATCGTACTATCTCG
TTTCAACATTATTACTAGACTTCCAAACTAAGCTATGCATACGAGGGAGTCTC
GTAAGTGTCGGAAGTCTTATAGAACGGGTTGTCTGTCTGTCGACAGGTGTCA
CAGCGAAAACAGTAAGATATATATCGAGATGTGCTTTTTTGCAATTGATTCA

GCGTTCCAAAGTTAGGTTCCGTCTTCTACCATAACTCTCTATTCAAACCGTAC
GTGGTTCAAATTAGTCATATCCGGTTTCGTCGATTATCGCCTTAGAACGTAGG
TAACAGTTAGTAAGAAGCATTTTTTTTTGTTTTGCAAACAATTCAGCGTCCCA
AGATAACATATCACGACTATTCACAACACTGTCAAAATCAGAAGTGGCACGA
ATTTGTTGTTACATCCGGTTTCAATACTGATTTCAGTAGAGCAAAAACGGTAA
ATGCTTTCAAGATGCAGTTTTCTCAAATCGATTCAGTGTTCCAAGAATAGTAT
TCACCTCCTCCCTTGGTAATCTATTCAAACAGAAAGTAGGTGAAACAAATGA
TTTTTGGTTTCAACCTTTATCACCATTGAACAAAACCAATAAGAGATTGAGA
GTAATGGCTTTTTGTTTTAGATTCAGATTTCGAGGGACAAGTTCTATCCTTGA
TTTTATTACTCCGTTGCAACCAGTACTTATAGATGCACTACTTCAAATTAGAG
TATTGAATTCTCATAGTTTGGAAGTCTTAGTGTCACACTGTGAAACGATACTC
ATTATTATTATGACAGAGACGTAAGCAAGGGGTAGGTCATGATGGGTCTGGA
TTCCCCCGAAATCCATACTTTTCTGTAAATTTAGTCTAATTTTTAGAGGTGAT
TTTACAAATTTTAATATAGTTTATCATCATATTGAATTTGAACTCCCCTCAAA
AGCTTTGGGCTCACCCAGAAATAAATTTCTGCATATGTGCCTGATAATTATTA
TTAACGTACAGAGAACTTTTGTGTACTTTCTAGGTCAGTTTGAAATGAAAGA
GATAGACATGTTTTTTTGAGATACTATTTTGAGACATGAACGAAAATGGACA
TGTTTTTTGAGATACTATTTTCTGATAAAGAAAGGAATGAATTGTAAGAATTA
ACCAGTATATAATATTTATCTAATAATATAATATATTTGATTACAGGTAACAA
CGTAGGCCAATGTGCTGATGGAAGCTACCAAATTGGATGCGGCACACAAGA
AACCTACAGTAACTGTGCTGATATCAGAATCAACTAA

Supplementary Note 2

List of non-redundant full length LPMO sequences from catalytic N-terminal histidine to the C-terminus, identified in the published transcriptome of *T. domestica*. The predicted protein sequences are named with the original contig identifier.

>GASN01405718.1
HGHLSVPPMRSSMWRDGYNVPPNYNDDGLNCGSFDVQWVKNGGKCGECGDD
YSLPRPRPNESGGMYGKNIIVANYQQGSTISVDLHIQAPHIGFFEFRLCARNDPNV
LETQDCFDQHLLELADGSGTRFTMEEHVAGEYTVDLKLPQGVTCTNCVLQWFW
RTGNRYGDCGDGTSGMGCGPQEEYRNCADIAIA

>GASN01404332.1
HGYMSNPPERSSAWRFGYDIPPNYNDNGLNCGGMGAQWDQNGGKCGVCGDD
YSLPRPRPNESGGTYGKGIIVGEYKAGQTISTTLTIVAPHIGWFEFRICARNDPNTL
EEQECFDQHLLELADGSGTRFTMTEHVAGDYVVDLKLPEGLTCTNCVLQWYW
RTGNRWGDCGDGTSGMGCGPQEEYRNCADISIS

>GASN01404421.1
HGHMADPPMRSSMWRYGFNTPPNYNDEGLNCGSFDVQWNQNNGMCGECGDD
YALSRPRPNESGGQYGTGIIVREYKAGSEVPILITIVAPHTGTFEFRVCPRNDPNV
LETQDCFDQHLLQLADGSGTQYYMPEHIAGNYTVNVKLPEGLTCTNCVLQWY
WRASNRWGPCGNGTSAGGCGPQETYRNCADVSIL

>GASN01011237.1
HGFMSEPPQRSSMWRSGFNTPPNYTDGSLNCGGFDVQWQQNDGKCGLCGDDY
ALAQPRPNESGGTYGTGVVGRTYKSGEVIEVSVNISASHVGWFEFHLCERNDPT
VMETEECFSQHILELADGSGTRYTLDNYNSGIRTVELQLPAGVTCTNCVFRWHW
QCGNRYGPCGDGTSAMGCGIQETYRNCADVAIE

>GASN01407914.1
HGHLADPPMRSSMWRVGFNTPPNYDDDGLNCGGFGVQWDQNNGLCGECGDN
YALPRPRPNEAGGKYGNGIIVRNYKAGEVIQVQLNFSTNHGGFSEFRLCPVNDP

KKLSTQECFDQHLLELADGSGTRYNHDEHQYPYVFGVKLPDGLTCTQCVFQWY
WKAGNRWGNCDDGTQGMGCGQQETYRNCADVSIA

>GASN01018175.1
HGFMSEPPERSSMWRAGFNVPPNYVDTGLNCGGYDVQWQQNGGKCGLCGDD
FALPQPRPNESGGTYGTGVIGRTYKSGEVIQVGVNISASHIGWFEFHLCERNDPN
VMETEECFAQHVLQLADGSGTRYPLSDYSPGIRNIELQLPAGVTCSNCILRWHW
ECGNRYGPCGDGTSAMGCGIQETYRNCADVAIE

>GASN01028018.1
HGYMSDPPMRSSMWRFGFNVPPNYNDNGLNCGGFGSNGGKCGVCGDPYNLAR
PRPNENGGKYGLGIIAKEYKEGQVVPVTLTIIANHVGYYEFRVCPLSQPNQLEEQ
SCFDQHLLELADGSGTKFHVPDSSGSEYTVEVKLPEGLTCDHCVLQWYWRTGT
FYGQCDDGTWAVGCGPQEIYYNCADISIS

>GASN01028062.1
HGYLIDPPQRSSMWRLGYDVPPNYYDMGLNCGSFPVMMANGGKCGICGDAYN
ITRPRPNETGGQYATGIIAAEYKQGQTISITIHMGANHKGWYEWRLCARNDPNQ
LEEQSCFDQHLLELADGSGTRYNQNDGTGNKEFVYELKLPAGVTCSHCVLQWY
WKAASYWGDCGDGTEGMGCGDQEIYINCADVSIS

>GASN01000264.1
HGFLSEPPERSSMWRFGFNVPPNYLDNALNCGSYGVQWYQNNGNCGICGDNY
ADPRPRPNENGGTYGLGIVGREYQEGQVVQVAINFTATHAGWMEWRICKLSDP
KELEEQECFDQNLLELADGSGTRYFLDQNDNNNHGVYTIDVRLPEGLTCDRCV
MQWYYRDGNRWGDCGNGSSAMGCGPQEEYRNCADIAIV

>GASN01028566.1
HGYMTDPAGRSTMWRFGFNVPPNYNDNGLNCGGFGVFMTNGGKCGICGDAY
NLPRPRPNESGGKYATGIIAREYKQGETIPVTLYMAANHKGWYEWRLCARNDP
YQLEEQECFDQHLLELADGSGTRYIQNDGTGNTNYTIQVQLPQGVTCSHCVLQ
WYWKAASYWGDCGDGTQGMGCGDQEVYINCADISIS

>GASN01400743.1

HGFMADPPMRSSMWRFGFNTPPNYADNELNCGGVGTQWAENGGKCGECGDP
YQLPRPRPNDNGGTYGRGVIAKEYTVGQVIPITINMSAAHLGYFEFRLCPVNSPT
VLETQECFDQHLLQLADGTGSKWKVPGGPGVYHIDVQLPSDLTCTQCVLQWYY
RTGNSWGTCDDGSQGLGCGPQETYSNCADIAIA

>GASN01024742.1
HGVLQEPPMRSLLWKFGYDTPPNYNPGGLNCGGYDAQWQEFNGECGECGDPIN
QPRPRENESGGKYGRGIIAREYTAGSTLRAVVDLYANHGGYFEFRLCPRNDPNV
LETQDCFDKYLLETVDGSTLYWTDGSISANYTVDLKLPKGVTCSNCVLQWYWR
CANRYGDCNNGTWSGGCGPQETYRNCADISIL

>GASN01009794.1
HGYQISPPGRSSMWRLGYGVPPNYYDMGLNCGSFPVMMANGGKCGVCGDAY
NITRPRPNETGGKYATGILAAEYKQGQTVPITLHMEANHKGWYEWRLCARNDP
NQLEEQSCFDQHLLQLADGSGTRYIQNDAMGNVNYTIPVNLPQGVTCSHCVLQ
WYWKAASYYGDCGDGTSAVGCGDQEIYINCADISIS

>GASN01000262.1
HGYMAEPPERSSMWRFGFNVPPNYNDNGLNCGTFGVQWQQNGGKCGVCGDN
YALPRPRPNENGGTYGLGIIGRTYKQGQTVQVAINFTATHAGWMEWRICKLSDP
KELEEQECFDQHLLELAXMMDQNDNNHQGVYTIDVKLPQGLTCDRCVMQWY
YHDGNQWGDCGNGSFGMGCGAQEEYRNCADIAIE

>GASN01404396.1
HGYMTVPPSRSSLWRFGYNSPANYNDMQLFCGGQGIQWGTNGGKCGECGDRY
DLPRPRPNESGGKYGTGIITENYKEGQVATFTVKLTTNHGGYFEFRLCPTNDPKK
LETQECFDQHLLELADGSGTQFHLPNRNSGEFNVDVKLPAGLTCSQCVVQWKY
VTATTWGQCDDGTSAAGCGPQETYQNCGDVSIE

>GASN01024272.1
HGYMEDPPARNAMWRFGYDTPVNWEDDELNCGSFGTQWDENGGKCGECGDA
YNLARPRPNETGGKFGTGIIVQKYTEGQVISVKVKLTTSHQGYFEFRLCPTNGEL
ETQECFDQHLLELADGSGTRYKVPLHEDGHGELTIDLQLPQGVTCEQCVLQWFY
RAGNNWGTCDDGSEGMGCGEQETYVNCADVSIS

>GASN01405771.1

HGYMADPPQRSSMWRFGFNTPINWEDDELNCGGIGTQWDQNGGKCGECGDAY
NLPRPRPNENGGKFGKGIIVKNYKAGEVTTVTITLTTSHQGYFEFRLCPTNGQEE
EQSCFDQHVLQLADGSGTKYYLPLYEGGEGDIEIDVKLPDGITCEKCVLQWFYR
AGNNWGTCDDGTEGMGCGPQETYVNCADISIS

>GASN02043256.1

HGYMSEPPQRSSMWRFGFNTPTNWNDDQLFCGGLSVQWNTNGGKCGECGDPY
NLPRPRPNEQGGEYGKGIIVRKYQAGQLIPVTITLHTSHQGFWEFRLCPTNGALE
TQECLDQHLLQLEDGSTQYHLPAQEGLSGEFNLNVRLPDNLTCEQCVLQWRWR
SGNNWGTCDDGTTGMGCGPQETYINCADISIS

>GASN01010363.1

HGYLVDPPNRSSLWRQGYDTPRNYNDNELFCGGYGVQWDQNDGKCGECGDP
YQLARPRPNEAGGQYGLGIITANYTAGQVATFTALLTTNHWGYFEFRLCPTNDP
TKLTTQECFDEYLLPLASGAGTQYHVPNHNNGQFNVHVKLPEGLTCSQCVVQW
RYVTATRWGDCGDGTSAMGCGPQEMFQNCADISIA

>GASN01405900.1

HGHMLEPPMRSSAWRFGYPIPPNYNDQGLTCGTYPIQWGQNDGKCGVCGDDY
ALPQPRPNENTGTYGLGVIVREYKEGQVVPIQINFTTTHGGYFQFNICPITVPGTIE
TDECFEQYPLELADGSGYKWKLSDMNIPGSYDLEVKLPEGLTCSQCVLRWHYN
AGHNWGDCGDGTGGMGCGAQEQTRNCADVTIS

>GASN01030700.1

HGHLHDPPSRNSAWHLGFNVPPTYNEDGLNCGGYGASGGKCGECGDPYADPRP
RAHENGGQFGTGVIVKEYIEGQTFTATVNWSTTHLGYNEFRLCPLKSDTELETQ
ECFDQYLLELADGSGTRYMLQENIVPVVHNIELKLPAGVKCEHCVLQWYWKAG
NNVGQCADGSYQIGCGTQETYSNCADIRIN

>GASN01404868.1

HGRLMDPPSRSSLWRFGFNTPINYDDNQLFCGGFQVQWEVNGGKCGICGDSWS
QTPPRDNELGGKYGSGIITRKYKSGSVITTTVQVTANHKGYFEFRVCPLNDSGTD

ATQDCLNQYQLPLANSGQYRYYLAPNVLGYINIPLQLPPDLICPHCVFQWHWNV
GNSWGNCGNGTSAVGCGPQETFRGCGDISIY

>GASN01008505.1
HGRLMDPPARNSMWRFGFPNPVNYNDNELFCGGWAVQWQQNRGQCGVCGDP
FHVREPRPHEAGGHYAKGIIGRHYSVGQVIDIEVELTANHWGRFELYLCPNNNP
RQVVTQECFDRYPLYVDGGNDVAFYIPYDSKKKVTFRYKVRLPPYVTCSQCVIQ
WTYYTGNMWGTCPNGTEAVGCGRPETFRNCADVRIVTSTGAFPPLGFQIDNPFL
LYYRDLRQPSKIQALVIRDQVCLPTQLYRRIPGMDQWCETNCLRYPPNCPSQLC
YCPQECDAIGELEGRFGADRYCQDECLVYGKKCPEHRCSCY

Supplementary Note 3

List of non-redundant full length LPMO sequences from catalytic N-terminal histidine to the C-terminus, identified in the transcriptome of *Lepisma* spp. (*de novo* assembled, see Methods for more details). The predicted protein sequences are named with the contig identifier.

>c79265_g1_i1
HGYMTAPPSRSSLWRFGFDAPANYNDMELFCGGQGIQWGTNGGKCGECGDNY
ALSRPRPNESGGQYGIGIITENYQQGQTATFSVKLTTNHGGYFEFRLCPTNDPKK
LETQECFDQHPLELADGSGSQFHLPNRNSGNFDVQVKLPAGLTCTQCVVQWRY
VTATTWGQCDDGSFASGCGPQETYQNCGDVSIQ

>c16949_g1_i1
HGHLADPPMRSSMWRVGYNTPPNYNDDGLNCGGFNVQWDQNSGLCGECGDD
YALPRPRPNEAGGQYGNGIIVRNYKAGEEIQVTLNFSTNHGGFSEFRLCPVNDPT
KLSTQDCFDQNLLQLADGSGTRYNHDQHQYPYVFGVKLPDGLTCTQCVFQWY
WKAGNRWGDCDDGTQGMGCGQQETYRNCADVSIA

>c42263_g1_i1_
HGFMSEPPERSSMWRAGFNVPPNYVDTGLNCGGFDVQWQQNDGNCGLCGDD
YALPQPRPNESGGTYGTGVIGREYKEGDIIQVGVNMSASHIGWFEFHLCARDDPS
VMETEDCFYQHVLELADGSGTRWPLSDYSPGIRNIEVQLPAGVTCSNCILRWHW
QCGNRYGPCDDGTSAMGCGIQETYRNCADVAIN

>c42263_g1_i2
HGFMSEPPERSSMWRAGFNVPPNYVDTGLNCGGFDVQWQQNDGNCGLCGDD
YALPQPRPNESGGTYGTGVIGREYKEGDIIQVGVNMSASHIGWFEFHLCARDDPS
VMETEDCFYQHVLELADGSGTRYPLDNYNSGIRTVELLLPAGVTCSNCVLRWH
WECGNRFGPCDDGTSAMGCGIQETYRNCADVAIN

>c45730_g1_i1
HGFMADPPMRSSMWRFGFNTPANYADNELNCGGVGTQWATNGGKCGECGDP
YNVARPRPNDNGGTYGTGVIAKEYTVGQVVPITINMSAAHLGYFEFRVCPVNDP

KKLETQECFDQHLLELADGSGTQWKVPSNPGVYHMEVQLPSDLKCTQCVLQW
YYRTGNSWGTCDDGSQGLGCGPQETYSNCADIAIQ

>c46276_g1_i1
HGYLEVPPQRSSMWRFGYDTPKNYEDDELNCGSFGTQWDQNGGKCGECGDAY
NLKRPRPNETGGRFGKGIIVANYTEGQVISVTVKITTSHQGFFEFRLCPTNGELET
QDCFDQHLLELADGSGTMYEMPYGEGGQGELQIDLQLPAGVTCKQCVLQWFY
RAGNNWGTCDDGSEGMGCGEQETYINCADISIS

>c46276_g2_i1
HGYLEVPPQRSSMWRFGYNTPRNYEDDELNCGSFGVQWDQNGGKCGECGDAY
NLARPRPNETGGKFGKGIIVANYTEGSVISVTVKVTVSHRGYFEFRLCPTNGELE
TQECFDQHLLELADGSGTRYKLKLKEDAHGQPQIDLQLPAGVRCEQCVLQWYY
RAANNWGTCDDGSEGMGCGEQESWANCADIAIL

>c46909_g1_i1
HGHMADPPMRSSMWRYGFSTPPNYNDEGLNCGSFDVQWGQNNGLCGECGDD
YGLSRPRPNESGGEYGTGIIVKEYKKGSEVPILITIVAPHIGYFEFRVCPRDDPSVL
ETQECFDQHLLQLADGSGTQYHMAEHVAGNYTATVKLPDDVTCTNCVLQWY
WRAGNRWGDCGNGTSAGGCGPQETYRNCADVAIL

>c48540_g1_i1
HGYQIDPPQRSSMWRLGYEVPPNYYDMGLNCGSFPVFMANGKQCGLCGDAYN
ITRPRPNETGGKYASGIIGKDYRQGQSVPITLHMEANHKGFYEWRLCARNDPNQ
LEEQSCFDQHLLELADGSGTRYIQTDGTGDTDYTIEVKLPQGVTCSHCVIQWYW
KAASYWGDCGDGTQGMGCGDQEVYYNCADISIS

>c48540_g1_i3
HGYQIDPPQRSSMWRLGYEVPPNYYDMGLNCGSFPVFMANGKQCGLCGDAYN
ITRPRPNETGGKYASGIIGKDYRQGQSVPITLHMEANHKGFYEWRLCARNDPNQ
WEEQSCFDQHLLPLSDGSGTRYVQNDAMGNVNYTISVDLPQGVTCSHCVLQW
YWKAASYYGDCGDGTSAVGCGDQETYYNCADISIS

>c49537_g1_i1

HGHLQEPASRNSAWHLGFNVPPTYNEDGLNCGGYGASGGKCGECGDAYSDPK
PRAHENGGEFGTGVIVKEYLEGQIITATVNWSTTHLGFNEFRVCPLKSDTEVETQ
ECFDQYLLELADGSGTRFMLEENIQPVVHEVQLKLPAGLKCDHCVFQWYWKAG
NNVGQCADGSYQLGCGTQETYSNCADISIS

>c50425_g1_i1
HGAMLDPPSRSVMWQFGYETEPNYNPGGLNCGGYDAQWQDFNGECGECGDPI
NMARPRENESGGKYGKGIVVKEYKQGSNIQVLVDLYANHGGYFEFRLCPRNDA
SELEQQDCFDKYLLETVDGSTQYYTEARDGNFTVNLKLPQGITCSQCVLQWYW
RCANRYGDCNNGTWSGGCGPQETYRNCADIAIQ

>c50623_g1_i1
HGNMLEPPMRSSAWRFGFAVPPNYNDQGLTCGTYPIQWGQNGGKCGVCGDDV
TIAQPRPNENTGTYGLGVIVREYTVGQVVPIQINFTTTHGGYFEFYLCPITVPGTIE
TEECFQQYPLPLADGSGYRWALADMNIPGSYNLEVQLPEGLTCSQCVLRWHYN
AGHNWGDCGDGTSAMGCGAQEQTRNCADVSIS

>c52749_g1_i1
HGHMQEPPMRSSMWRYGFNTPPNYNDDELNCGGMSVMWEQNSGECGECGDA
YNLARPRPNEAGGKYGNGIVVREYTVGETIPVQLNFSTTHAGYFEFRLCPTNDP
KKLSTQDCFDQNLLEVADGGTRWTLPDNNQAGFYNLNLKLPDGVSCSFCVLQ
WLYVAGNRWGDCGDGTSGMGCGQQETYRNCADISIS

>c56329_g1_i1
HGHMSAPPMRSSMWRFGYDVPPNYNDDGLNCGGYDTEWIQNGGKCGECGDD
YSLARPRPNESGGTYGKNIIVETYSEGATISVTLTIQAPHIGYFEFRLCQRDDPTVL
ETQDCFDQHLLELADGSGTRFVMEEHVAGDYTVDLKLPAGVTCKACVLQWFW
RSGNRYGDCGDGTSAVGCGPQEEYRNCADVAIA

>c56329_g1_i2
HGHMSAPPMRSSMWRFGYDVPPNYNDDGLNCGGYDTEWIQNGGKCGECGDD
YSLARPRPNESGGTYGKNIIVETYSEGATISVTLTIQAPHIGYFEFRLCQRDDPTVL
ETQDCFDQHLLELADGSGTRFVMEEHVAGDYTVDLKLPAGVTCKACVLQWFW
RSGNRYGDCGDGTSAMGCGPQEEYRNCADVAIA

>c59140_g2_i1

HGYLINPAQRSSMWRLGYDVPPNYYDMGLNCGTFDVFMANGRKCGLCGDAY
NMTRPRPNETGGQYATGILAGEFKQGQTMSITLHMGANHKGFYEWRLCARNDP
NEWEEQECFDQHLLQLADGSGTRYNQMDDTGDKDFIIDVKLPDGVTCSHCVLQ
WYWKAGHYWGDCGDGTSGMGCGDQETYYNCADISIS

>c132113_g1_i1

HGYMAVPPMRSSMWRFGYDTPINWEDDELNCGGIGTQWDQNGGKCGECGDA
YNLARPRPNENGGKFGKGIIVAEYKAGEVITVTITLTTSHQGYFEFRLCPTNGEPE
EQSCFDQHVLQLADGSGTQYHLPNEEGAEGDINIDVKLPDDITCEKCVLQWFYR
AGNNWGDCGDGTEGMGCGPQETYENCADITIS

>c24288_g1_i1_c3217_g1_i1

HGRLMDPPARNSMWRFGFPNPVNYNDNELFCGGWAVQWQQNRGQCGVCGDP
YHVREPRPHEAGGHYAKGIIGRHYSVGQVIDVEIELTANHWGRFELYLCPNNNP
RQVVTQSCFDRYPLYVDGGNDVAFYIPYDSKKKVTFRYKVRLPPYVTCSQCVIQ
WTYFTGNMWGTCPNGTEAVGCGRPETFRNCADVRIITSTGAFPPLGYQLDNPFL
LYYRDFRQPSKVQALVIRDQVCLPTQLYRRIPGMDQWCETNCLRYPPNCPSQLC
YCPQECDAIGELEGRFGADRYCQDECLVYGKKCPAHRCRCY

Supplementary Note 4

List of non-redundant full length LPMO sequences from catalytic N-terminal histidine to the C-terminus, identified in the genome of *D. melanogaster*. The original gene annotation symbols (according to FlyBase) and the proposed new nomenclature (in brackets) are shown at the front of each protein sequence.

>CG42749 (*DmAA15A*)
HGRLMDPPARNAMWRFGYPNPVNYNDNELFCGGYAVQWEQNKGRCGICGDA
YHVKSPRPHEAGGQYAKGIISRYYTAGQTIDVEVELTANHYGRFEMFLCPNNNP
RQEATQQCFDRYPLLISGSREHRYLIPRDAKKKDIFRYKVRLPPYVTCTQCVLQW
TYYTANMWGTCANGTEAVGCGKAETFRNCADVAIVSNTGGGIPPPFVNNKSPY
LLYYRDYRAPADNNIFPLIVRDQKCIGAPAFRALPGIDNWCEINCLRYPPNCPED
ACQCPQECVAIGEYAGQDGADTYCMDKCLNYESECPPDRCRCY

>CG4362 (*Dm*AA15B)
HGMMLSPPSRSSRWRYDGSAPQNWNDNELFCGGLYTQSNNGGRCGLCGDNFL
DAQPRANEIGGSIGGAGVVTRSYVAGNTITVGVKITTNHLGYFEFHLCNLDAFG
AESEECFDQNRLRFIDGSDRKDIGDQMGEFDVTVVLPEGLTCSHCVLRWTYVGA
NNWGICDNSGNGALGCGPQETFKNCADVSIYWGRNLVKELVGGGDPVAPVEV
A

>CG4367 (*Dm*AA15C)
HGMMLSPTGRSSRWRYDNSAPTNYDDNALYCGGFWKQTENDGKCGLCGDDW
SLEQPRPNELGGKYGSGVIVKSFAGVDEAEINVKITANHLGYFRFHICDLDENGS
ESEDCFNQYPLNFTDGSQKYYINTTTGDIPVTVKLPSDLNCIHCVLRWTYTAGNN
WGVCEDGTGAMGCGAQETFINCADISVLSSARSIIQEVPVEVAESK

>CG42598 (*Dm*AA15D)
HGRLVEPPGRASAWRFGFQTPPDYNDHELNCGGLSRQWQRNGGKCGECGDAW
DLPEPRPHEYGGHWGKGQIVRSYLPGSQMTIRVELTASHMGYFEFRICPNPNAK
QSCLDENVLSILNGSPSQPNESDLDTRFYPRNGSCIYEILAQLPDFTCEHCVLQWR
YVAGNNWGMCGNGIGAIGCGPQEEFRSCSDIALTTEYLHPYLSPINPPPSQSNGM

SANTKHNTVNIQSYKLKYICIILLLLILILVCVVVMSIKFHNHNSFCIPQFCNNQKK
YLFWNSSQHNNFICDLFKKNITLSNDKLDDPVHKLGD

>CG41284 (*Dm*AA15E)
HGRLVEPPGRASAWRFGFQTPPDYNDHELNCGGLSRQWQRYGGKCGECGDAW
DLPEPRPHEYGGHWGKGQIVRSYLPGSQMTIRVELTASHMGYFEFRICPNPNAK
QSCLDENVLSILNGSPSQPNESDLDTRFYPRNGSCIYEILAQLPDFTCEHCVLQWR
YVAGNNWGMCGNGIGAIGCGPQEEFRSCSDIALTTEYLHPYLSPINPPPSQSNGM
SANTKHNTVNIQSYKLKYICIILLLLILILVCVVVMSIKFHNHNSFCIPQFCNNQKK
YLFWNSSQHNNFICDLFKKNITLSNDKLDDPVHKLGD

Supplementary Note 5

List of non-redundant full length LPMO sequences from catalytic N-terminal histidine to the C-terminus, identified in the genome of *T. castaneum*. The original gene annotation symbols (according to iBeetle) are shown at the front of each protein sequence.

>TC016344
HGMLMEPPNRSSLWRFNPNAPVNYDDDQNYCGGMNVQWDQFGGKCGVCGD
QYDDPHPQANENTGKYGQGIIAKEYTAGSVIDVQVVLTTNHMGYFNFSLCVLED
PNAPESGEECFKPITLGDGSDKYVLPNTEEEDNDTINTTVKLPDGLTCDRCVLRW
TYTAGNNWGQCEDGSYDEGCGPQETFRSCTDIAIH

>TC016345
HGMMLEPPNRSSLWRFNSSAPINYNDNQNFCGGFSVQWGKFGGKCGPCGDKY
DDPHPQANENGGKYGRGFVVAEYKAGSVIDVQVKLTANHLGYFKYSLCVLKD
PNGPEMDEKCFMPLKLVDGSVKYNVWKTDYLIKNKLKLPTKIKCDRCVLRWN
YRTGNNWGNCGNGTSGLGCGPQETFRSCSDVRIV

>TC016346
HGMMLQPPNRSSLWRFNPNAPPNYNDNQNYCGGAGVQWASLGGKCGLCGDK
YDDPHPQANENTGKYGQGIIAAEYKAGSAIDVEVLLTTNHKGFFNFSLCVLQNP
NAPESGEDCFQPLKLANGDKQYNVVTGEKTINTKVQLPSGLTCDRCVLRWHYQ
AGNNWGQCEDGSYDQGCGPQETFRSCADVTIS

>TC016347
HGMMLEPPNRSSLWRFDPTAPVNYNDNQNYCGGASFQWQSMDGKCGVCGDP
YNAPHPQENENTGKYGQGKIVRQYSPGSVVDIQVSLTTNHLGYFLFSVCVLQDP
SAPESGEECFQPISLANGDDRYNVTFSERTVNTQVKLPDGLTCDRCVLRWHYIG
GNNWGQCEDGSYQEGCGPQENFRSCADVAIL

>TC016348
HGMMMEPPNRSSLWRFDPTAPPNYDDNQNFCGGVAVQWKQFNGKCGVCGDI
YDAPHPQDNENTGTYGQGKVVRTYNSGSVVDITINLTANHMGYFNFSVCVLQD

PNAPESGEECFQPITLANGEPRYYIQSTDKTLIVDTQVKLPDGLKCDRCVLRWHY
NCGNSWGQCDDGSYAEGCGPQETFRSCADVAIV

>TC016349
HGMMLEPPNRSSLWRYDKSALPNYQDNQNFCGGYYVQWELNGGRCGVCGDT
YSDPHPQDNENSGKYGSGKIVRTYEAGSVINVDVWLSKNHLGSFEFSLCEITNPN
APESGEECFKVLPLADGSQQYNVSAGETDITVALQLPKGKSCAHCVLRWHYRA
GNNWGDCGDGTWAKGCGSQETFRTCADVAIV

>TC016350
HGMMLEPSNRASLWRFDWKQPSNYNDMGYFCGGVKFGLVTIATMVSKFVTVV
LALVCLQKVSGHGMLMDPTNRASRWRVDPKAPINYDDNAFFCGGFAVQYEQN
GGKCGVCGDNYADPVPRSNENTGKFGNGVISKTYTAGSIITANVTLTANHLGSF
SYSLCVLKDPTKPETEDCFVDLPLADGSSKYPVSADEYEIVNQVQLPAGVTCDR
CVLRWHYKSGNSWGICTDGTQRMGCGAQETFRSCADIAIVA

>TC002263
HGRLMDPPARNSMWRFGFPNPVNYNDNELFCGGYAVQWEQNNGKCGLCGDP
HHVKEPRPHEAGGLYAKGIISRHYSVGQEIDIEVELTANHYGRFEIFLCPNNNPN
QVATQDCFDRYPLYLSGTRNFVYNIPEDGKKKAIFRYKVQLPPYVTCTQCVLQW
SYYTGNQWGTCPNGTEAQGCGKSETFRNCADVAIHTSAGSAVPPLFVGVNNPY
LLYYKDFSKPAPYNVYPLVVREQVCVPNSLYKSIPGVNEWCQSNCLRYPPNCPA
KICQCPTTCEAIGEYEGHPGADVQCMDECLVYPSKCPLDRCFCYEEFSTN

>TC015490
HGRLIDPPSRASAWRYGFDTPHNYNDHELYCGGFTRQWVKNEGKCGVCGDAW
DSKIPRAHEFGGTYGQGVIVRKYTAGSVINIRVELTANHFGYFTFAVCPDFKRAT
QKCLDKHVLKLVKPQEGVDHHHSTRYYPKEGNKVYEMKYRLPKATCDHCLFQ
WRYIAGNNWGTCPNGTGAVGCGPQEEFRACADVTISGKGVQEMEEDTTEVEVE
VATTTVAAPPSGEETHSPITALVLSLVSFLLVFLILSLLYIHFYQVGKQLKSWLKG
DKDKEQAPMPPPRTRRARNVDGLDEVDLKVESVA

Supplementary Note 6

List of non-redundant full length LPMO sequences from catalytic N-terminal histidine to the C-terminus, identified in the genome of *A. gambiae*. The original gene annotation symbol (according to VectorBase) and the proposed new nomenclature (in brackets) are shown at the front of each protein sequence.

>AGAP001819
HGMALDPIARGSRWRCNPSALPNYTDNELFCGGFQVQWGTHNGKCGVCGDNY
GDARPRLHELGGPFGPGDIVRQYVRGSVIEARIRLTANHRGYFYFDLCNLDVGG
AEDEACFSQYPLSLADGSRNWVLPSTAVGEYRINLKLPDSLTCSHCIFRWTYVA
GNNWGYCEDGSGRLGCGPQETFKTCSDVRIVASNDINPSFAYCTKAV

>AGAP000266
HGRLMEPPARNAMWRFGFPNPVNYNDNELFCGGYAVQWEQNQGNCGVCGDA
YHLRAPRPHEAGGEYGKGIVSRRYVAGQELEVEIELTANHMGRFELYLCPNNNP
RAEATQDCFDRYPLYLSGTREVRFFIPPDSKKKDVFRYRVQLPLYVSCTQCVLQ
WTYFTGNMWGRCDNGTESVGCGRPETFRNCADISIVSNTGGGRPPLFVGNNNPF
LLYYRDFRDPKPDNVYPLIIRDQVCLPTATYRSFIGMEEWCQSNCLRYPPNCPET
VCHCPQTCEAIGELRGREGADVYCLDQCLNFKSNCPADRCRCY

>AGAP001705
HGRLIEPPSRASAWRYGFSTPPNYNDHELYCGGFNRQWQRNEGKCGECGDPFD
APLPRPHEFGGKWGQGVIVRRYKPGTTITLRVELTASHMGYFEFRICDNVQAKQ
DCLDKHLMRIVSGTPSIPHPNDLKTRFYPRNGSRIYDMKAELPADLNCNNCVVQ
WKYVAGNNWGICPDGNGAVGCGPQEEFRACADVSVSEKDGQDNRTPLRPSQK
PTPTRAPDSSTEPAEGEQPIEEEPPKGVKYMGPLVAIISLFLVLCGFAALYIYHYH
GGRIKALMRWNREKTQKGAHGTEPIANMGGAGVAHHGDVEANAPVPPPRTKRI
SNQIRDIDAQESSVLSGSTKRQSPLDITVSRQPSGMD

Supplementary Note 7

List of non-redundant full length LPMO sequences from catalytic N-terminal histidine to the C-terminus, identified in the genome of *H. azteca*. The accession identifiers are shown at the front of each protein sequence.

>XP_018015775.1
HGRLLDPPARGTMWRLGFQTPPDYNDHELFCGGFHRQWTTNGGRCGECGDPW
DLPRPRPHEAGGVFGTGTLSRIYRQGQIITAVVHLTANHFGWFEFRLCPTNNPNE
YAKQSCLNQNILELADSPGTRFVIEDQSQVLFRVRLKLPRGLKCSHCVLQWHYT
SGNNWGFCKDGNDMMGCGKQEIFRGCSDVAIFDQSDSTYYKHLNLTKDFDFTP
IINLQDGTYMRKHQSGEEILAVKGQPAPESNSDENQNEILDKESRVENAKDDLSQ
LDVDGDGVINIDGNLLSLDALKNHLMSSGLLTESPGANSHNEQEDSELQSGFQV
SSSTSRSRSKFRADRTKEDKKTNIITTSKKRRNYATLNTDIKTQNLGKPSDSVDII
NSAYISSNTFRKSDQSSHSKYTTTEPPVAPVQYATESSNIAEYTLGPVVVHRPARP
KAEHSGFNDFPSASSEKSSYMDTSSSHSVLAKPFVPEPEDVLPEILSQLTSLKDFPI
QKHTSSNDAANAHFISTTDQTRLLEAVKSMFSASQATTPSPATQLHRIESQDNTV
SIPNIIVSPQSNEMVSFGFRRKPTSPDDHTFPAGIFSTITETATKSSRKAKPMPQAR
QSRPRTATPQTLLDVQATIRTLAAQTQLFSKLNPDDKSREPKPLSSLSTDESIASH
TVWKSKPKSSSVRMSKIETSPVRHERPNSLVTRAPLTDLTRHRDLPEPNLSSASR
TPFSAIASQALFSQVPDMAGAMAFQVPLSKLRDTATLDRLLELKMSLLPEDERQ
AHLDPVLLIIM

>XP_018009620.1
HGRLIEPPSRASAWRFGFSTPRDYNDNEGFCGGKAKQHDVNGGRCGVCGDSWE
LQPRPHEVGGLYATGIIVRNYSTGQVIEATAQITANHKGFFEFRICPTNDPSIEATQ
DCLDRYPLYGVGSSDHRLYIGHNNGNHSFRLRLPQQLNCRHCVLQWRYVAGN
DWGICYDGSGALGCGPQEEFRACADVSISADRGSHPAREARRRTTHASTNPRRR
VTVAPVDDAPQDDVVLTQTGRACEARGIWRAVPGMHAWCTTNCNHTPAFCPT
SHCWCT

>XP_018007939.1
HGRLMDPIARNAMWRAGFPNPVNYNDNELYCGGFVVQYQQNGGKCGVCGDV
WTEKPPRSHEAGGLFGNAIIAKRYTSGQVIDIEAELTTNHMGNMEVRLCPNNDP

DAAITEDCFEKYLLGLADGSDSVFVIPEGTKKSEIFRWKVKLPEDVTCSHCVIRW
KYFAGNTWGKCDNGSESVGCGNQETFINCADVIINSNTATAAATSDFNPWALYS
SRDNVVQNVSAEEAAQQGLKPLIIRAQRCIPIDPFHNVANMDMWCMINCLKYPP
NCHPSYCKCVHDCSAVGEFALIEDADIWCHQNCLKHPSYCPPERCHCV

>XP_018028131.1
HGRLIQPPARSTAWRYGFPTPVNYNDHETYCGGYGRQWGVNRGKCGPCGDPW
DMPQPRDNEEGGKYGTGVITATYKEGAEVELGVELTANHQGFFEFRLCPNNNP
WYYPPPGTRKMYMRYALPSGLTCTQCVLQWRYVAASNWGDCGNGTGRMGCG
PQEEFRACADISIISDDGTANSSPAAPVDVTVSPVDEDYNEVDTGRLHEQEAHQL
QESNYIGHVVALTLAFVLLPLLLLALVIYFYFAREKVRKFVRKNLDCWKKTVGG
VTVPEGPLDGVDPGLLEAKNPNRSSLEFPNHPYGAAPTAPPRRNRGRSPSVSSNR
GEEQSASPNPV

>XP_018020658.1
HVALMEPPARNVLWRAGYPNLPKHEDDDYLYCIEKEEKNCPPCGDTIDSPQPYA
HQAGGKWALGIIGRNYSAGETISVGVNVTNSHGGHMVFKLCPNNDVTKPVTQE
CLDRYPLSASGYPDGKVPVTSPYQETQEFNLKIKLPQDVVCSQCVLQMTQYTEQ
FKPAKVMFRNCADIGIIDPRSASSHARQNRFASTSKSLTNGFTQQGFLDQSAGFG
LAGQQQFRTALQEPSSFLTIADQLSAYGGSPQFGQLAFDNHSPQVQSSSVHSQTP
TFAYEPDFMNGQFDFKSALEFNNQQSLQQVGDGNNRENFQLPSRPEFDIINSGNP
QVAAANKQDQKFKTAKFLSASAPQQFSSLSPLSPQVPFTRTIQGTENFSKFQNSK
QISHQIGAPFVQQKPQAVEIYGFGNVGKKQKNNAKRDSDANFAPSSSDSQFRSRS
KAMFPEQGSQQSPGYILAHIY
