# Supplementary material for: An ancient family of lytic polysaccharide monooxygenases with roles in arthropod development and biomass digestion
Source: Nat Commun. 2018 Feb 22;9:756. doi: 10.1038/s41467-018-03142-x (PMC5823890; doi:10.1038/s41467-018-03142-x)
Supplement: Supplementary file 3 — Description of Additional Supplementary Files [file 41467_2018_3142_MOESM3_ESM.pdf]

Description of Additional Supplementary Files

File Name: Supplementary Data 1
Description: List of the 100 most abundant proteins identified in the proteomics of the crop fluids from Thermobia grown on Avicel. The table includes the best blastX hits against NCBInr, E-value, top blastX species and accession, predicted CAZy family and molar percentage abundance calculated from emPAI values obtained from shotgun proteomics. In order to increase the stringency of the analysis, only contigs with 2 or more significant peptide matches were considered.

File Name: Supplementary Data 2
Description: List of the 100 most abundant proteins identified in the proteomics of the crop fluids from Thermobia grown on wheat straw. The table includes the best blastX hits against NCBInr, E-value, top blastX species and accession, predicted CAZy family and molar percentage abundance calculated from emPAI values obtained from shotgun proteomics. In order to increase the stringency of the analysis, only contigs with 2 or more significant peptide matches were considered.

File Name: Supplementary Data 3
Description: List of the 100 most abundant proteins identified in the proteomics of the crop fluids from Thermobia grown on filter paper. The table includes the best blastX hits against NCBInr, E-value, top blastX species and accession, predicted CAZy family and molar percentage abundance calculated from emPAI values obtained from shotgun proteomics. In order to increase the stringency of the analysis, only contigs with 2 or more significant peptide matches were considered.

File Name: Supplementary Data 4
Description: List of the 100 most abundant proteins identified in the proteomics of the crop fluids from Thermobia grown on oats. The table includes the best blastX hits against NCBInr, E-value, top blastX species and accession, predicted CAZy family and molar percentage abundance calculated from emPAI values obtained from shotgun proteomics. In order to increase the stringency of the analysis, only contigs with 2 or more significant peptide matches were considered.

File Name: Supplementary Data 5
Description: Hit values obtained by searching LPMO protein sequences against the hidden Markov model (HMM) created from the multiple sequence alignment of AA15s.
